# Sequencing the transcriptome of milk production: milk trumps mammary tissue

**DOI:** 10.1186/1471-2164-14-872

**Published:** 2013-12-12

**Authors:** Danielle G Lemay, Russell C Hovey, Stella R Hartono, Katie Hinde, Jennifer T Smilowitz, Frank Ventimiglia, Kimberli A Schmidt, Joyce WS Lee, Alma Islas-Trejo, Pedro Ivo Silva, Ian Korf, Juan F Medrano, Peter A Barry, J Bruce German

**Affiliations:** 1 Genome Center, University of California Davis, 451 Health Sciences Dr, Davis, CA 95616, USA; 2 Department of Animal Science, University of California Davis, One Shields Ave, Davis, CA 95616, USA; 3 Department of Human Evolutionary Biology, Harvard University, Peabody Museum, 11 Divinity Avenue, Cambridge, MA 02138, USA; 4 Department of Food Science and Technology, University of California Davis, One Shields Ave, Davis, CA 95616, USA; 5 California National Primate Research Center, University of California Davis, Road 98 and Hutchison Drive, Davis, CA, USA; 6 Center for Comparative Medicine, University of California Davis, One Shields Ave, Davis, CA 95616, USA

**Keywords:** Mammary gland, Milk, Rhesus macaque, Human, Transcriptome, RNA-Seq, Milk RNA, Bioinformatics, Milk processing, Transcriptomics

## Abstract

**Background:**

Studies of normal human mammary gland development and function have mostly relied on cell culture, limited surgical specimens, and rodent models. Although RNA extracted from human milk has been used to assay the mammary transcriptome non-invasively, this assay has not been adequately validated in primates. Thus, the objectives of the current study were to assess the suitability of lactating rhesus macaques as a model for lactating humans and to determine whether RNA extracted from milk fractions is representative of RNA extracted from mammary tissue for the purpose of studying the transcriptome of milk-producing cells.

**Results:**

We confirmed that macaque milk contains cytoplasmic crescents and that ample high-quality RNA can be obtained for sequencing. Using RNA sequencing, RNA extracted from macaque milk fat and milk cell fractions more accurately represented RNA from mammary epithelial cells (cells that produce milk) than did RNA from whole mammary tissue. Mammary epithelium-specific transcripts were more abundant in macaque milk fat, whereas adipose or stroma-specific transcripts were more abundant in mammary tissue. Functional analyses confirmed the validity of milk as a source of RNA from milk-producing mammary epithelial cells.

**Conclusions:**

RNA extracted from the milk fat during lactation accurately portrayed the RNA profile of milk-producing mammary epithelial cells in a non-human primate. However, this sample type clearly requires protocols that minimize RNA degradation. Overall, we validated the use of RNA extracted from human and macaque milk and provided evidence to support the use of lactating macaques as a model for human lactation.

## Background

Most studies of mammary gland biology utilize cell culture and rodent models. Although human mammary tissues have been accessed via oncological or breast reduction surgery, access to normal tissue during lactation is extremely rare. Therefore, genome-wide transcriptomic analyses of the lactation cycle have been mainly limited to rodents and agricultural domesticated livestock [1-18].

We and others [19-21] recently reported a novel, non-invasive technique for the *in vivo* study of mammary epithelial cells from humans based on the isolation of RNA from cytoplasmic crescents of milk fat globules. Mammary epithelial cells are the cells of the mammary gland that produce milk. Mammary epithelial cells secrete fat into milk by an apocrine mechanism that occasionally traps portions of cytoplasm within the membrane of the milk fat globule [22,23]. Thus, the RNA within the milk fat layer is expected to represent RNA from mammary epithelial cells at the time they are producing milk. With the advent of next generation sequencing technologies, it has become possible to catalog all RNAs inside the cytoplasmic crescents of milk fat globules. However, the transcript profile of cytoplasmic crescent RNA has never been directly compared with that for RNA derived from the mammary gland epithelium in the same primate species. Maningat et al. [20] compared their microarray results derived from human milk cytoplasmic crescent RNA with 98 previously reported transcripts in the whole mammary glands of lactating mice. Brenaut et al. [24] compared microarray results from the cytoplasmic crescent RNA and mammary tissue in goats. While these two studies provide some reassurance, the validity of milk-derived RNA in humans would be more appropriately obtained in a lactating primate model. Thus, our first objectives were to assess the presence of cytoplasmic crescents in the milk of rhesus macaques and the ability to obtain sequence-quality RNA from macaque milk fat. Our next objective was to compare RNA from macaque milk fat with RNA from mammary biopsy tissue from the same animals to determine whether RNA from milk fat can be used to non-invasively study the transcriptome of milk production in primates.

The incidence of cytoplasmic crescent formation varies by species, milking interval, and time of day [22,23]. Cattle have the lowest incidence of crescent formation. Instead of extracting RNA from milk fat, another approach to access the transcript profile of milk-producing cells has been to probe the RNA of somatic cells in milk from cows [16-18]. Despite the fact that mammary epithelial cells are a minority cell population in milk [25], the authors reported informative transcriptomes using this method [16-18] and extended it to humans [26]. They also compared the transcriptomes derived from a sample of cells in milk and a mammary biopsy sample from a Holstein cow and found that 84.5% of genes shared expression between the two sample types [15]. Thus, an additional objective of the current study was to determine the extendibility of these results to primates by comparing RNA extracted from macaque milk cell fractions with mammary-derived RNA.

There are various reasons to validate a non-invasive method for sampling the mammary gland, especially in humans. Most breast cancers arise from epithelial cells; these cells sampled via milk could potentially be used to identify cancer biomarkers [27]. A diagnostic approach during lactation is urgently needed given the transient increase in the risk of breast cancer post-partum precisely at a time when women cannot be evaluated by conventional methods [28]. Another use for non-invasive sampling of milk-producing cells in humans is in the study and diagnosis of lactating women with insufficient milk supply. Only 13.3% of women in the U.S. exclusively breastfeed for 6 months post-partum, well below the national goal outlined in Healthy People 2010, with insufficient milk supply noted as the primary reason for premature weaning [29]. An understanding of the biological basis of insufficient milk supply would enable improved diagnosis and treatment.

Given that biopsy is an extremely invasive approach for the study of normal mammary biology in humans, especially during lactation, we investigated whether RNA isolated from milk is representative of RNA from the mammary gland in a lactating, non-human primate model (*Macaca mulatta*). We first determined the effects of milk collection and processing on RNA quality and origin (e.g. exosomal or cellular) in both rhesus macaques and humans. We then compared paired samples of RNA extracted from milk fat, cells in milk, and mammary tissue at two stages of lactation in rhesus macaques.

## Methods

### Human subjects

Five healthy multiparous lactating mothers who had recently given birth to sons were recruited via flyers placed in the Northern California communities of Davis, Sacramento, and Vacaville. Due to sex biases in milk composition [30], mothers were selected for the study only if they were nursing male infants. Exclusion criteria also included smoking, primiparity, pre-term birth, multiple birth, metabolic disease, intention to breastfeed for less than six months, or planning to take oral contraceptives prior to six months post-partum. The UC Davis Institutional Review Board approved all aspects of the study and informed written consent was obtained from all participants. This trial was registered on clinicaltrials.gov (ClinicalTrials.gov Identifier: NCT01817127).

### Human milk collection

Milk was collected twice from each participant—once at 90 days and once at 180 days post-partum. On the day of collection, participants were instructed to use a breast pump to empty one breast between 7 am and 9 am and then collect milk from that same breast 4 hr later. Fresh milk samples were immediately transported to the laboratory on ice.

### Animal selection

Rhesus macaques were selected from among animals in the outdoor breeding colony at the California National Primate Research Center. Inclusion criteria also included prime-aged mothers (~7–10 yr old) with at least three prior pregnancies, nursing male infants less than 1 mo old. All aspects of our study were approved by the Animal Care and Use Committee at the University of California, Davis.

### Milk collected from macaques

Milk samples were collected twice from each macaque mother during lactation, once at 30 days post-partum and once at 90 days post-partum, using procedures previously described [30]. On the day of milk collection, the mother-infant dyads were transported to temporary indoor housing. Mothers were lightly sedated and placed in mesh jackets (ProMed-Tec, Inc. Bellingham, MA) to prevent nursing and to allow milk accumulation for 3.5 to 4 hr. Infants were housed with their mothers during the period of milk accumulation. After milk accumulation, mothers were sedated (ketamine, 5–15 mg/kg) and administered oxytocin to stimulate milk let down (2 IU/kg). Milking was performed by gently hand stripping the nipples to mimic infant nursing behavior as previously described [30].

At an additional time point between 30 and 90 days post-partum, milk samples were collected from three of the macaques using a repeated milking protocol. After a 2-hr milk accumulation period, the mothers were sedated, administered a half-dose of oxytocin (1 IU/kg), and milked as noted above. They were then administered oxytocin again (1 IU/kg) exactly 10 min after the first dose and were milked a second time.

### Macaque mammary biopsies

Six macaques underwent a single vacuum-assisted core biopsy of the mammary gland in accordance with guidelines set forth by the University of California, Davis Animal Care and Use Committee. Three of the macaque mothers were biopsied 30 days post-partum and the other three were biopsied 90 days post-partum. Immediately following milk collection, mothers scheduled for biopsy were further sedated with dexmedetomadine (15 mcg/kg, intramuscular). The surgical site was prepared aseptically and blocked with lidocaine. Biopsies were performed using the Suros ATEC biopsy system as previously described [31], and the incision site closed by suturing. Biopsied dams were then housed indoors with their infants and monitored for adverse signs. Analgesia (ketoprofen and/or buprenex) was administered at the discretion of the staff veterinarian. One of the six macaques required post-biopsy antibiotic therapy. All macaques were returned to their outdoor corrals after the biopsy site had healed (approximately 5–7 days).

### Milk processing

Whole milk samples were centrifuged at 2311 *g* for 10 min at 4°C. The fat layer was collected using a sterile spatula and transferred to TRIzol. To collect RNA from cells in milk, the skim supernatant was removed and TRIzol added to the PBS-washed cell pellet. All samples were stored at -80°C until RNA extraction. Detailed protocols for the processing of human and macaque milk are provided in Additional file 1.

### RNA extraction and assessment

RNA was extracted using the Qiagen Universal Kit (Qiagen, Netherlands), followed by treatment with DNAse. RNA yield and quality (260/280 nm and 260/230 nm, respectively) were measured by Nanodrop spectrophotometry (Thermo Fisher Scientific, Inc., Wilmington, DE). RNA integrity was assessed with an Agilent Bioanalyzer (Agilent Technologies, Santa Clara, CA).

### Milk imaging and analysis

Slides containing whole milk that had been incubated with 0.1% acridine orange (AO) (4) were viewed on an Olympus VS110 whole slide scanner. Three images were captured—a differential interference contrast (DIC) image to view the fat globules, a fluorescence channel for AO-RNA, and a second fluorescence channel for AO-DNA. Images were analyzed using our Globulator software [32], which was developed to identify sizes and locations of milk fat globules, cytoplasmic crescents, and nucleated cells, and to compute summary statistics. The Globulator is a Perl program that uses ImageJ [33] to process the stained slides, to differentiate between globules and crescents, and to compute statistics. This software first identifies milk fat globules and crescent location independently by color. The milk fat globule locations and area are then corrected and each crescent is linked with the closest milk fat globule, generating a list of (X, Y) coordinates and area of each globule, crescent, and linked crescent-globule. A description of this method is provided in Additional file 2. A 300 MB image containing 20,000 globules can be processed in under a minute and multiple images can be processed in batch. The accuracy of this method was confirmed by manually comparing a subset of processed and original images.

### RNA library construction and sequencing

RNA extracted from macaque milk fat, macaque milk cells, and macaque mammary tissue was used to create libraries for RNA sequencing (RNA-Seq). Total RNA was purified, fragmented and converted to cDNA, and then ligated to adapters. The cDNA products were purified and amplified by PCR to create a library for subsequent cluster generation using the TruSeq RNA Sample Preparation kit (Illumina, San Diego, CA) with pPoly(A) selection. RNA-Seq libraries were multiplexed in batches of 12 and sequenced on an Illumina HiSeq 2000 Sequencing System in a single read 100 bp format.

### RNA-Seq data processing

FASTQ files were de-multiplexed to assign the 100 bp reads to the originating sample. Reads were first trimmed to remove adaptor sequences using Scythe [34], and then adaptively trimmed for quality on both the 5′ and 3′ ends using Sickle [35]. Resulting reads were checked for quality using FastQC [36]. The rhesus macaque genome assembly rheMac2 was indexed using Bowtie [37]. Reads were then mapped to the indexed genome using TopHat [19289445] [38]. Transcript abundances were estimated as fragments per kilobase of exon per million fragments mapped (FPKMs) using Cufflinks [39] and all MMUL1.0 Ensembl transcripts from the Ensembl database, release 67 [40]. The data have been deposited at NCBI’s Gene Expression Omnibus [41] and are accessible through GEO Series accession number GSE49765 [42].

### RNA-Seq analysis

To identify differentially expressed genes, counts for each gene were first calculated by applying HTSeq-count [43] in “intersection-nonempty” mode to the mapped reads (e.g. TopHat output) after preparation with Samtools [44]. The R package, DESeq [45], was used to apply a variance stabilizing transformation to the count data. To determine differentially expressed genes, the nbinom test function within DESeq was used to test the significance of the difference between the base means of two conditions (e.g. milk fat vs. mammary biopsy, day 30 vs. day 90). Differences were considered statistically significant if *p* < 0.05 after adjustment for multiple hypothesis testing using the method of Benjamini and Hochberg (method = “BH”) [46].

### Functional enrichment analysis

Human orthologs of the macaque Ensembl transcripts were identified using Ensembl tools and custom scripts. When a macaque transcript mapped to multiple human orthologs, the ortholog with the highest sequence identity was used. Biological functions of gene sets of interest were then determined using the Functional Annotation tools within DAVID Bioinformatics Resources [47]. All Ensembl IDs were used as the background list. Functional annotations were considered statistically enriched when *p* < 0.05 after adjustment for multiple hypothesis testing using the method of Benjamini and Hochberg (method = “Benjamini”) [46].

## Results

### Sources of RNA in milk

**Figure 1:**
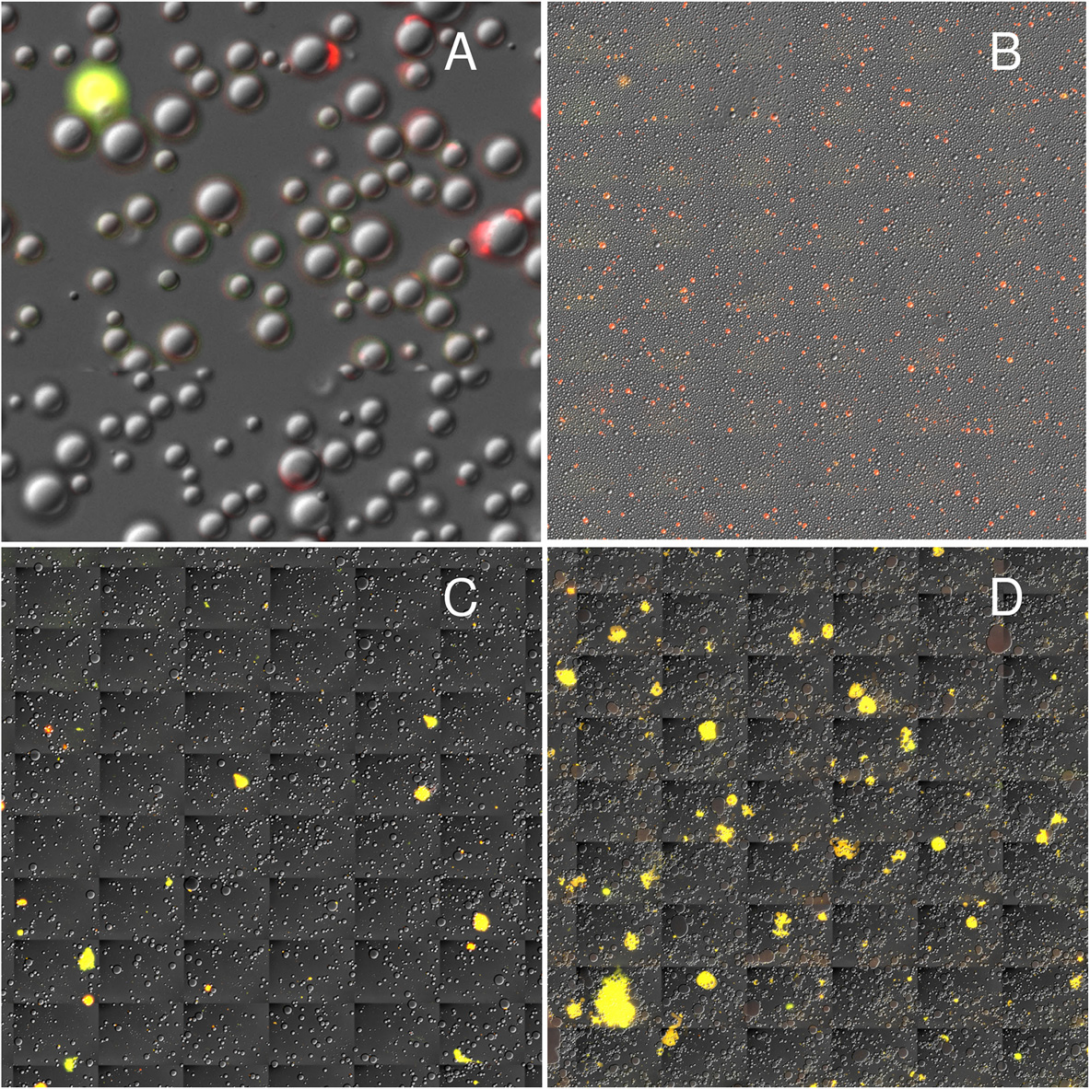
**Composite images of whole milk stained with acridine orange (AO).** Each image contains three channels: 1) a differential interference contrast image to view the fat globules, which look like gray bubbles; 2) a fluorescence channel for AO-RNA; and 3) another fluorescence channel for AO-DNA. When AO associates with RNA, the emission maximum is 650 nm (red). When AO associates with DNA, the emission maximum is 525 nm (green). Therefore, areas containing only RNA (e.g. crescents) look red, only DNA look green, and both RNA and DNA (e.g. nucleated cells) look yellow. **(A)** Close-up of human whole milk with a nucleated cell (yellow) and several crescents of various sizes. **(B)** Whole slide scanned image of human whole milk collected after a 4-hr milk accumulation. **(C–D)** Whole slide scanned images of macaque whole milk after **(C)** a 4-hr and **(D)** a 10-min milk accumulation.

Whole milk contains both cells and cytoplasmic crescents, which are sources of cellular and exosomal RNA, respectively. The proportion of cellular to exosomal RNA in milk is unknown. Therefore, to distinguish the exosomal RNA within the cytoplasmic crescents from RNA within nucleated cells, we developed a method to quantify milk fat globules, cytoplasmic crescent RNA, and cellular RNA in whole milk stained with AO. Three channels—a differential interference contrast (DIC) image, a fluorescence channel to capture AO-RNA, and a second fluorescence channel to capture AO-DNA—were used to generate images using an Olympus VS110 whole-slide scanner. The DIC image shows the locations of the milk fat globules and the overlay of the AO-RNA, and AO-DNA channels indicate whether the RNA is exosomal (no DNA) or cellular (both DNA and RNA). An example of the resulting image is shown in Figure 1.

Samples of fresh whole milk from three human participants were evaluated for the percentage of milk fat globules with crescents and the percentage of whole milk RNA estimated to be from nucleated cells. In milk samples from three human participants, crescents were associated with 5.34%, 7.34%, and 5.50% of milk fat globules. In these same milk samples, 21.5%, 14.2%, and 0.8% of the whole milk RNA, respectively, was estimated to originate from nucleated cells origin. We additionally evaluated RNA within the milk fat and skim fractions of milk from one of the human participants. The whole milk contained 14.1% cellular RNA. When centrifuged into fat and skim fractions, the RNA attributable to nucleated cells in the milk fat fraction dropped to 8.7%.

**Figure 2:**
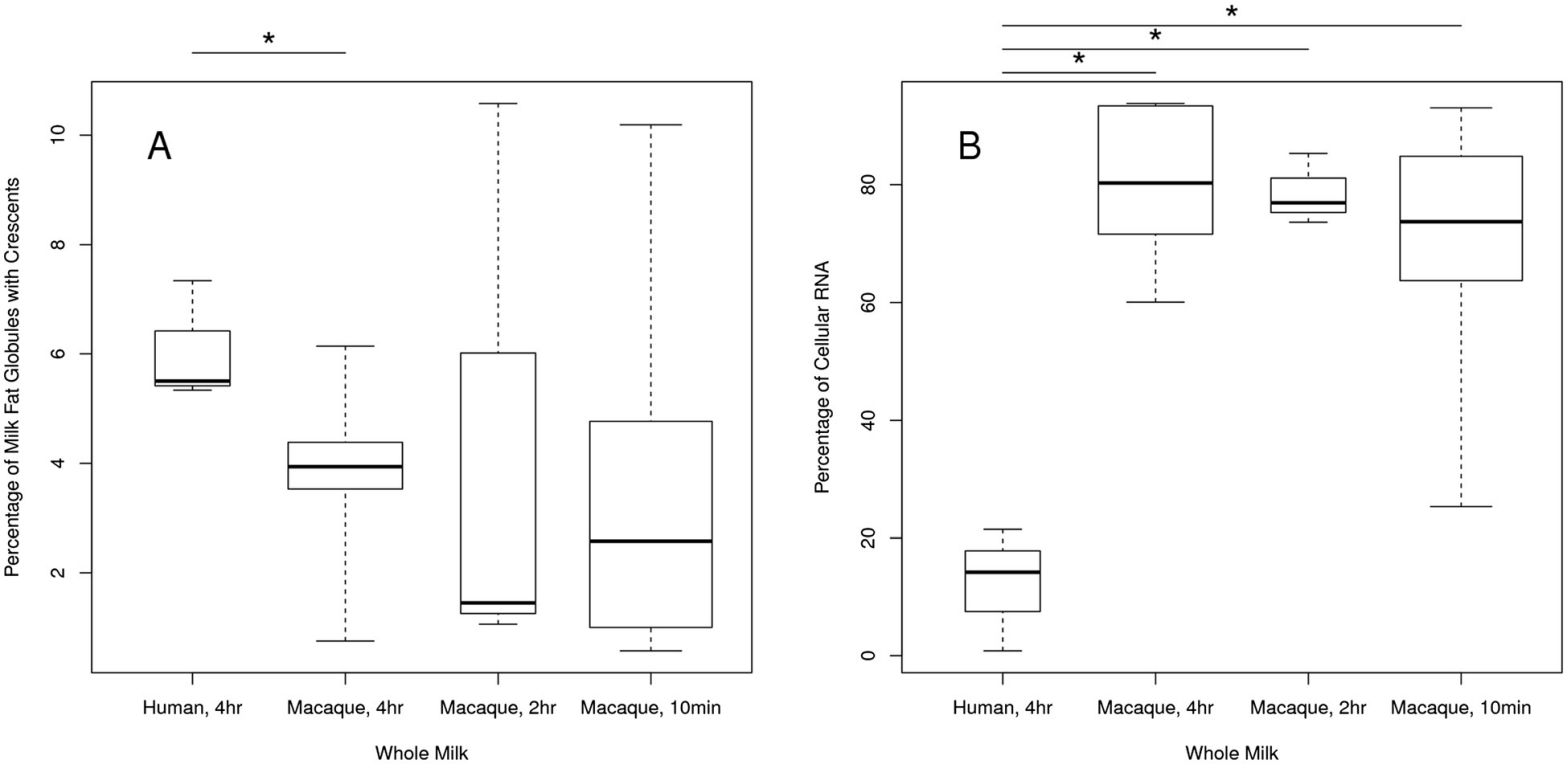
**Percentage of cytoplasmic crescents and cells in milk.** Milk samples were collected after a 4-hr accumulation period in human subjects and a 4-hr, 2-hr, or 10-min accumulation in macaques. The boxplots show the **(A)** percentage of milk fat globules with cytoplasmic crescents and **(B)** the percentage of RNA attributable to nucleated cells in these milk samples. **(A-B)** The upper and lower bounds of each box denote the first and third quartiles of the data, the dark horizontal line within each box denotes the median value, and the whiskers extend to the minimum and maximum values. The width of each box is proportional to the square root of the samples size. Asterisks denote statistical significance (*p* < = 0.05).

Fresh milk was collected from six macaques at various stages of lactation and after different periods of milk accumulation (10 min, 2 hr, or 4 hr) to determine the incidence of cytoplasmic crescents and nucleated cells. The percentage of globules with crescents in whole milk was significantly different between humans and macaques after 4 hr of milk accumulation (*p* = 0.046), but not after 2 hr or 10 min (Figure 2A). The percentage of RNA in whole milk attributed to nucleated cells was higher in macaques regardless of the period of milk accumulation (*p* < 0.01) (Figure 2B). The repeated milking protocol (10-min accumulation) did not significantly affect the proportion of RNA attributable to crescents or nucleated cells. In summary, the percentage of RNA attributable to cytoplasmic crescents was moderately lower and nucleated cells was significantly higher in whole milk from macaques compared with humans. The milk of both species clearly contained both exosomal and cellular RNA.

### Integrity of RNA in milk

**Figure 3:**
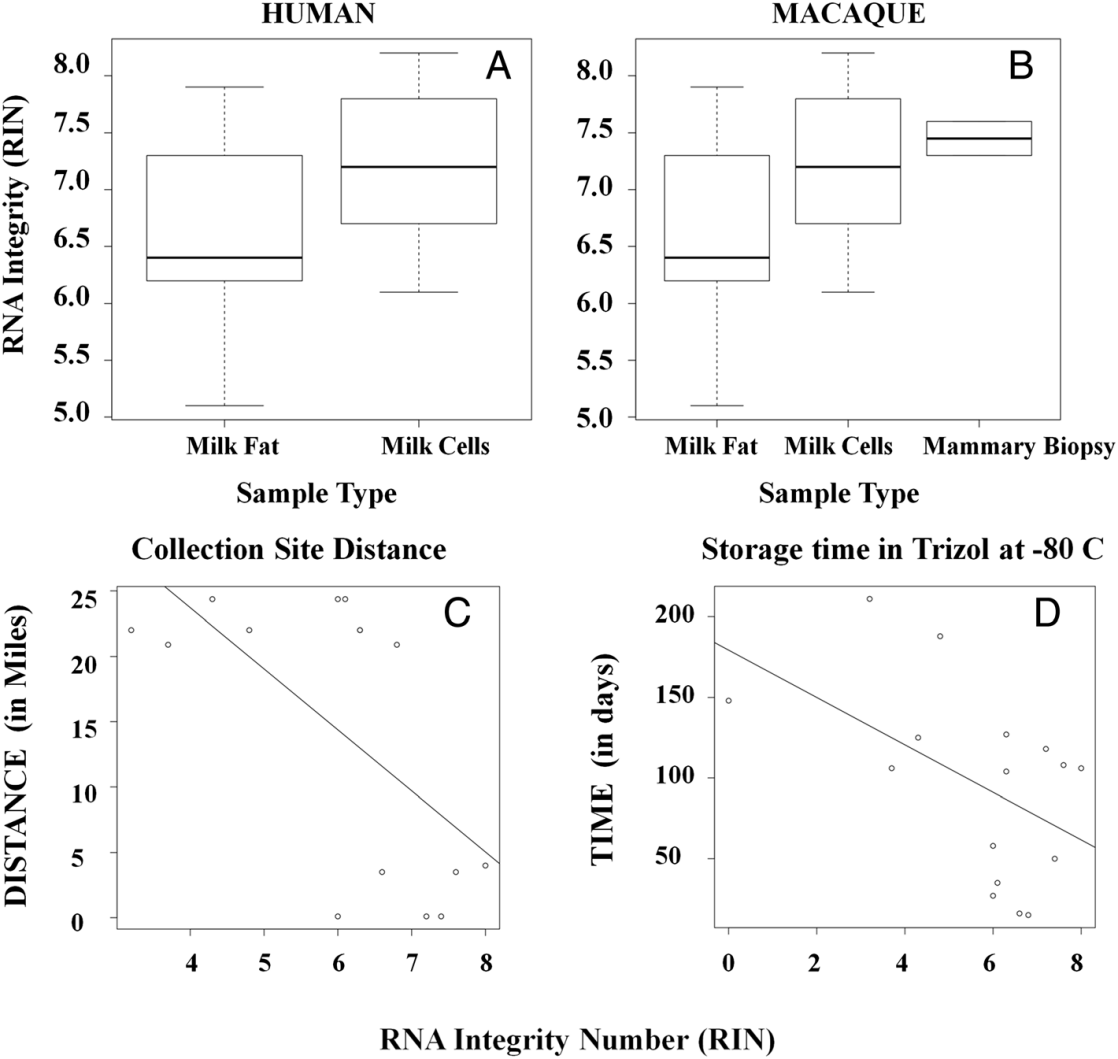
**Effect of milk fraction and processing time on RNA integrity. (A-B)** RNA degradation by sample types in **(A)** human and **(B)** macaque samples. All differences are significant (*t*-test, *p* < = 0.05). **(C-D)** Human milk fat RNA degradation by processing time in terms of **(C)** the distance to the lab from the site of collection (*R*^*2*^ = 0.40, *p* = 0.012) and **(D)** the amount of storage time in TRIzol at -80°C prior to RNA isolation (*R*^*2*^ = 0.26, *p* = 0.044).

The utility of a milk-based assay of gene expression in milk-producing cells is limited by the ability to acquire abundant RNA of sufficient quality for RNA sequencing. We therefore determined what factors, if any, affected the integrity of RNA isolated from human and macaque milk fat and milk cells. RNA Integrity Numbers (RINs) for total RNA extracted from the milk fat were lower than those for RNA extracted from the cells in milk (human, *p* < 0.01; macaque *p* = 0.032) or mammary tissue (macaque *p* < 0.01) (Figure 3A, B). In the human, but not the macaque samples, the RINs for total RNA derived from the human milk fat were negatively associated with processing time. RINs were also negatively correlated with the sample transport time (distance from the collection site to the laboratory; *R*^*2*^ = 0.40, *p* = 0.012) and with the time stored in Trizol at -80°C (*R*^*2*^ = 0.26, *p* = 0.044) (Figure 3C, D). RINs were not significantly affected by milk accumulation time in macaques; there was no difference between 10 min or 4 hr of accumulation. Overall, RNA from human milk fat was more vulnerable to degradation compared with other sample types and was negatively affected by processing delays prior to RNA purification, whereas RNA from other sample types was relatively stable.

### Biopsies of mammary tissue from lactating macaques

**Figure 4:**
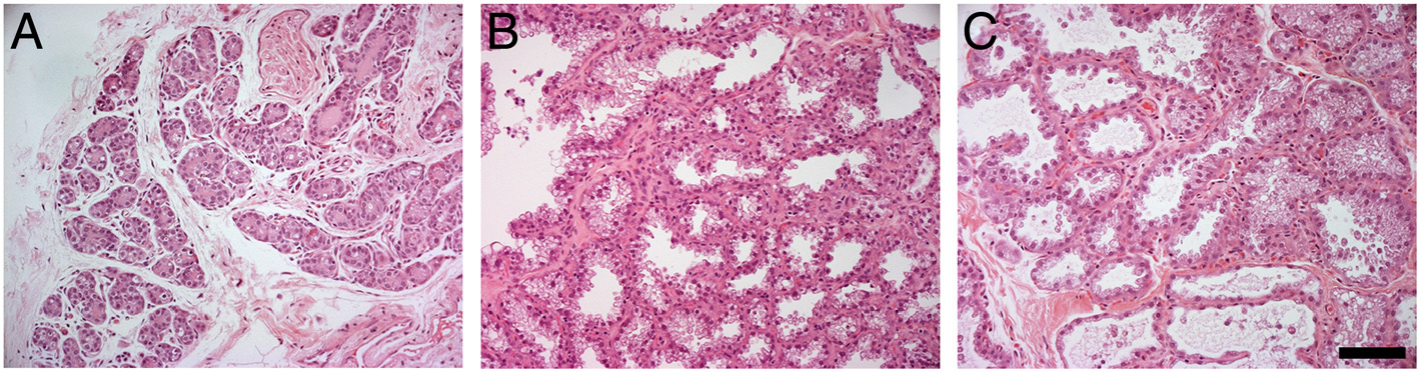
**Hematoxylin- and eosin-stained mammary biopsy samples from rhesus macaques.** Histology of tissue biopsied from the mammary glands of rhesus macaques at different stages of lactation. Sections (4 μm) were stained with hematoxylin and eosin. Scale bar = 100 μm. **(A)** Multiparous aged female macaque, non-pregnant, non-lactating; **(B)** multiparous female macaque, lactation day 30; and **(C)** multiparous female macaque, lactation day 90.

Mammary tissue from each of the six macaques was obtained by biopsy at one of the two time points (day 30 or day 90 of lactation) for comparison with RNA from milk sampled at the same time points. The only complication that arose from using this approach to sample the mammary glands of lactating rhesus macaques was that one female developed mastitis that was resolved by antibiotic treatment. At biopsy a portion of the tissue was preserved and stained with hematoxylin and eosin. Examples of biopsies from three life stages are shown in Figure 4. Histological analysis of the tissue collected from lactating dams confirmed the presence of lobulo-alveolar secretory epithelium comparable to that described for the human breast.

### RNA-Seq of milk and mammary transcriptomes

Of the 36 macaque samples, 34 were selected for sequencing—6 mammary biopsy samples (one from each animal), 6 milk cell samples from day 30, 6 milk cell samples from day 90, 4 milk fat samples from day 30, 6 milk fat samples from day 90, and 6 cell-free milk samples from day 90. The RNA extracted from the day 30 milk of two of the macaques was judged to be of insufficient quality for sequencing (RIN < 6).

RNA-Seq libraries were constructed and sequenced for all 34 samples. More than 1.5 billion reads were mapped to the macaque genome with an average of 45.9 million reads obtained per sample. Gene expression intensity was normalized to FPKM and summarized at the transcript level for each sample. A summary of FPKMs for all transcripts in each sample is provided in Additional file 3.

### Cell-specific markers in milk and mammary transcriptomes

**Figure 5:**
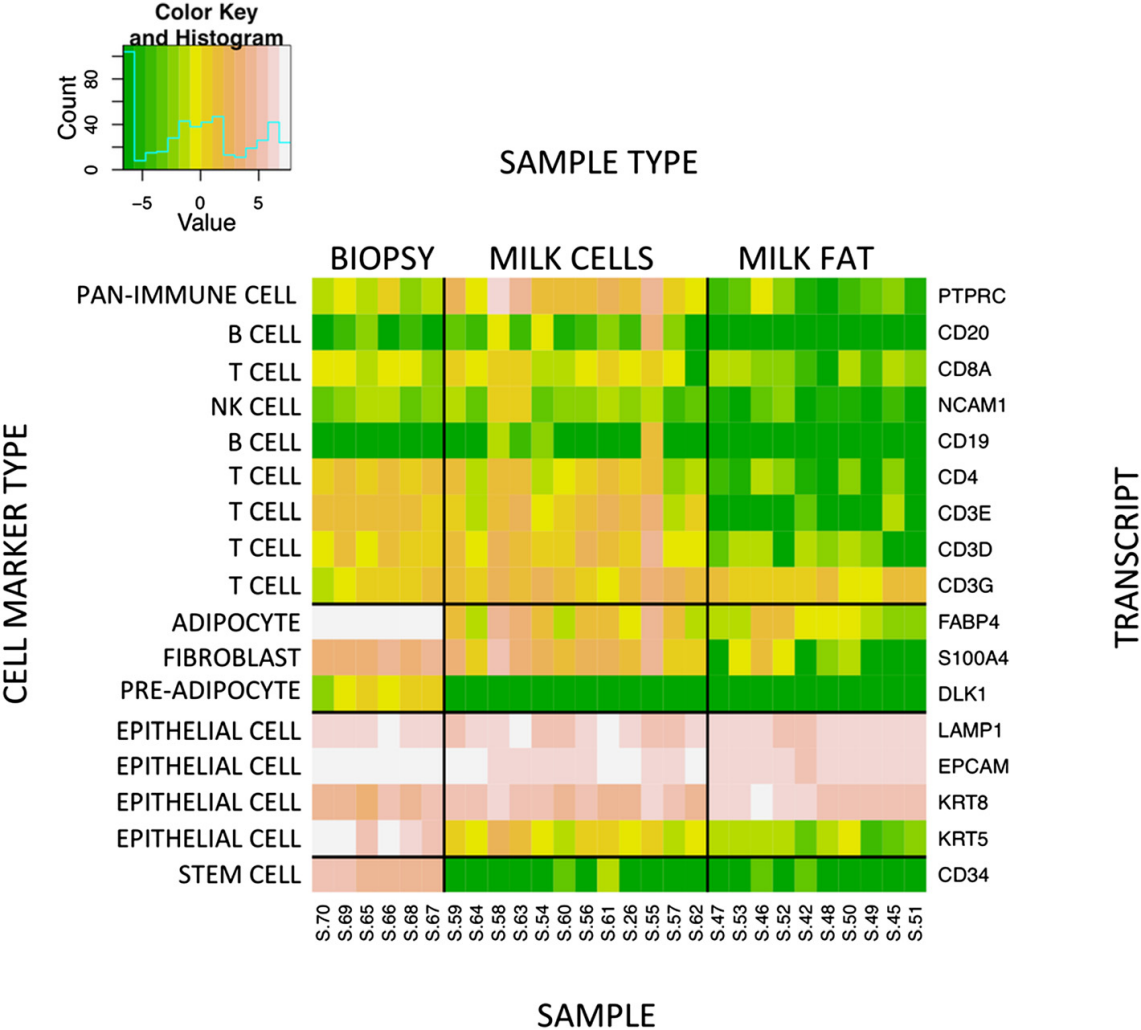
**Expression of cell-specific markers in milk and mammary transcriptomes.** Each box in the heatmap represents log-transformed expression intensity. The color key indicates the level of expression: Green = low/no expression, Yellow = low expression, Orange = moderate expression, Pink = high expression, and White = very high expression. The color key also includes a light blue line that indicates the number of observations at each level of expression.

Mammary tissue consists of mammary epithelial cells as well as stromal and immune cells. Milk fat fractions are hypothesized to contain exosomal RNA that originate in milk-producing cells. Milk cell fractions are hypothesized to contain a heterogenous mixture of cells. To test these hypotheses, we examined the genetic expression of cell-specific markers using the RNA-Seq data obtained from macaque milk fat, macaque milk cells, and macaque mammary tissue (Figure 5). Transcripts corresponding to immune cell markers were most abundantly expressed in the milk cell fraction, were moderately expressed in mammary tissue, and were minimally present in milk fat. As expected, gene expression signatures associated with stromal cells were most abundant in mammary tissue. Not surprisingly, a gene expression signature for mammary epithelial cells was present in all samples. Milk-producing cells specifically express cytokeratin 8 (KRT8), but not cytokeratin 5 (KRT5), whereas basal mammary epithelial cells are KRT5+/KRT8- [48]. The milk fat fraction clearly contained RNA from KRT5-/KRT8+ cells while the mammary biopsy and milk cell fractions clearly contained RNA from other epithelial cell types (Figure 5).

**Figure 6:**
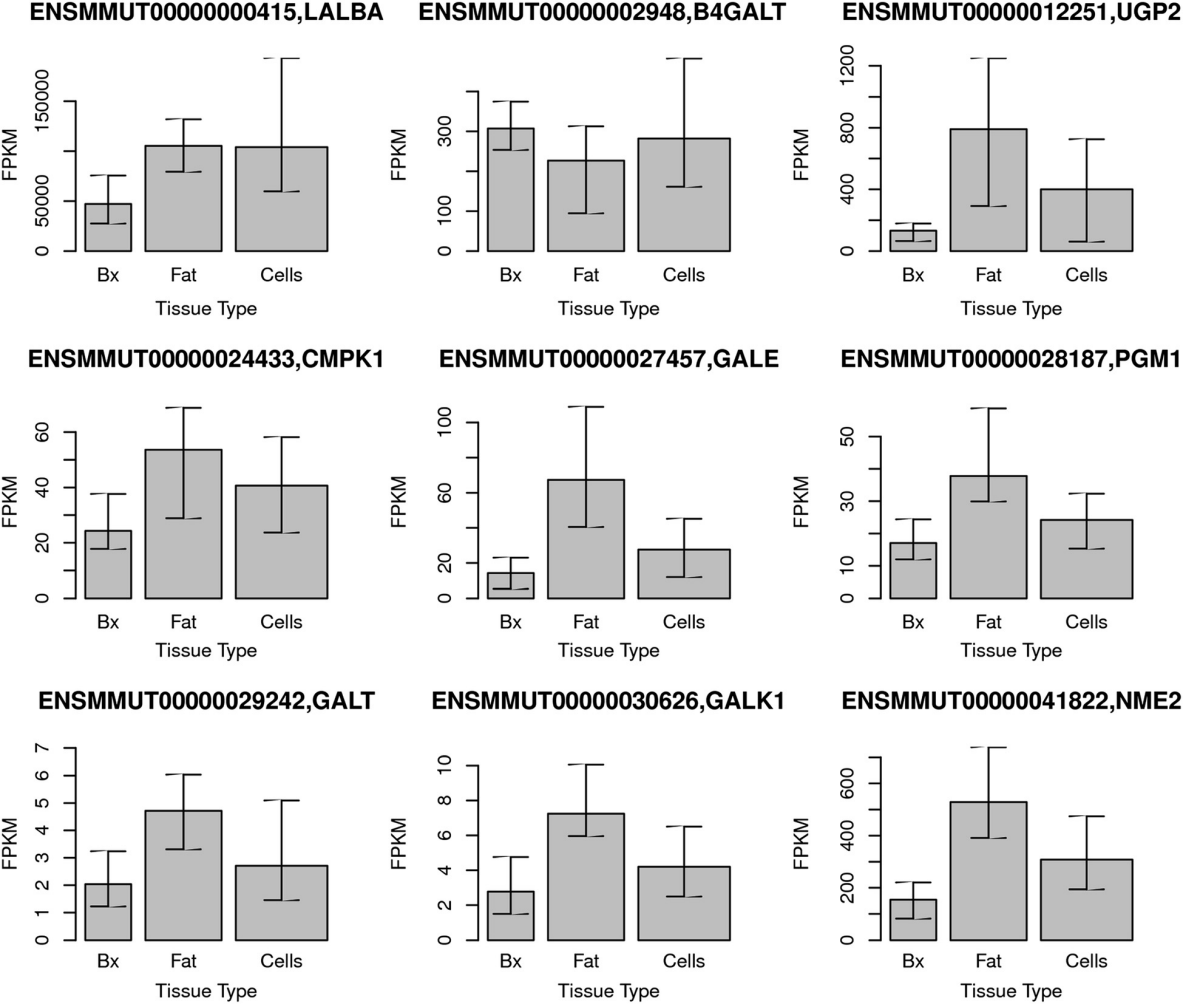
**Abundances of transcripts that code for proteins in the lactose synthesis pathway.** All differences between mammary biopsy (“Bx”) and milk fat samples (“Fat”) were significant. Differences between milk fat (“Fat”) and milk cell (“Cells”) samples were significant for all transcripts except LALBA and B4GALT.

In order to verify the specificity of the milk fat fraction for milk-producing cells, we next examined a process unique to milk-producing cells—lactose biosynthesis. We compared the key genes involved in lactose synthesis (LALBA, SLC2A1, B4GALT1, SLC2A9, UGP2, CMPK1, GALE, PGM1, GALT, GALK1, CANT1, NME2, HK1, and SLC35A2 [21]) among the three sample types. Of these 14 genes, the expression of 8 was significantly higher in RNA from milk fat compared with mammary biopsies, one was significantly lower, and 5 were not different (Figure 6). Compared with milk cell samples, transcripts for eight of the lactose synthesis genes were more abundant (*p* < 0.05) in milk fat and the other six were not different. Thus, most transcripts associated with lactose synthesis were more abundant in RNA isolated from milk fat compared with RNA from cells in milk or mammary tissue.

### Highly expressed genes in milk and mammary transcriptomes

In each of the three sample types—macaque milk fat, macaque milk cells, and macaque mammary tissue—the transcript pool was dominated by the expression of just a few genes (Tables 1, 2 and 3). Transcripts corresponding to the abundant milk proteins CSN2, CSN3, LGB2, and LALBA were the most abundant in all three sample types. Together, the top 20 most abundant transcripts accounted for 70–80% of all mRNA transcripts in the samples (biopsy, 79.4%; milk cells, 74.6%; milk fat, 70.8%).

**Table 1:** Top 20 Transcripts in macaque mammary biopsies

**Symbol**	**Description**	**Bx mean**	**Cells mean**	**Fat_mean**
CSN2	Casein beta	490,313.83	380,205.92	287,623.70
CSN3	Casein kappa	84,657.12	58,584.42	46,774.06
LGB2	Beta-Lactoglobulin-2	59,847.85	73,933.99	79,475.38
LALBA	Alpha-Lactalbumin	47,105.07	104,098.11	105,391.85
CSN2	Casein beta	41,747.42	60,781.56	57,631.93
FABP3	Fatty acid binding protein 3	19,808.37	19,580.82	33,508.00
LYZ	Lysozyme	18,660.12	39,052.05	34,365.67
LTF	Lactotransferrin	8,142.20	4,414.17	3,811.85
CD24	CD24 molecule	4,273.60	6,136.86	5,046.04
ncRNA	Non-coding RNA	3,098.27	3,392.18	1,018.07
B2M	Beta-2-Microglobulin	2,963.40	3,644.58	1,486.42
TPT1	Tumor protein, translationally-controlled 1	2,736.16	2,347.90	2,982.91
FTL	Ferritin, light polypeptide	2,715.15	4,262.47	2,882.36
GLYCAM1	Glycosylation dependent cell adhesion molecule 1	2,187.31	2,878.29	4,108.55
MT1E	Metallothionein 1E	1,873.84	2,184.34	1,810.93
SPP1	Secreted phosphoprotein 1	1,816.97	2,764.59	1,780.25
FTH1	Ferritin, heavy polypeptide	1,774.86	2,505.45	3,398.50
SERF2	Small EDRK-rich factor 2	1,573.25	1,892.39	3,066.99
ATP5J2	ATP synthase, H + transporting, mitochondrial F0 complex, subunit F2	1,371.16	1,269.64	1,874.09
ncRNA	Non-coding RNA	1,176.23	5,467.23	16,500.49

**Table 2:** Top 20 Transcripts in macaque milk fat

**Symbol**	**Description**	**Bx mean**	**Cells mean**	**Fat mean**
CSN2	Casein beta	490,313.83	380,205.92	287,623.70
LALBA	Alpha-Lactalbumin	47,105.07	104,098.11	105,391.85
LGB2	Beta-Lactoglobulin-2	59,847.85	73,933.99	79,475.38
CSN2	Casein beta	41,747.42	60,781.56	57,631.93
CSN3	Casein kappa	84,657.12	58,584.42	46,774.06
PLIN2	Perilipin 2	475.05	1,118.13	42,229.07
PLIN2	Perilipin 2	863.64	1,769.06	37,946.93
LYZ	Lysozme	18,660.12	39,052.05	34,365.67
FABP3	Fatty acid binding protein 3	19,808.37	19,580.82	33,508.00
PLIN2	Perilipin 2	254.88	617.99	17,045.39
ncRNA	Non-coding RNA	1,176.23	5,467.23	16,500.49
ncRNA	Non-coding RNA	685.90	5,428.94	16,401.81
ncRNA	Non-coding RNA	582.53	3,137.07	14,389.45
ncRNA	Non-coding RNA	459.06	2,539.38	7,970.79
PLIN2	Perilipin 2	115.13	285.20	6,184.76
CD24	CD24 molecule	4,273.60	6,136.86	5,046.04
GLYCAM1	Glycosylation dependent cell adhesion molecule 1	2,187.31	2,878.29	4,108.55
ncRNA	Non-coding RNA	86.23	943.72	3,907.15
LTF	Lactotransferrin	8,142.20	4,414.17	3,811.85
FTH1	Ferritin, heavy polypeptide	1,774.86	2,505.45	3,398.50

**Table 3:** Top 20 transcripts in macaque milk cells

**Symbol**	**Description**	**Bx mean**	**Cells mean**	**Fat mean**
CSN2	Casein beta	490,313.83	380,205.92	287,623.70
LALBA	Alpha-Lactalbumin	47,105.07	104,098.11	105,391.85
LGB2	Beta-Lactoglobulin-2	59,847.85	73,933.99	79,475.38
CSN2	Casein beta	41,747.42	60,781.56	57,631.93
CSN3	Casein kappa	84,657.12	58,584.42	46,774.06
LYZ	Lysozyme	18,660.12	39,052.05	34,365.67
FABP3	Fatty acid binding protein 3	19,808.37	19,580.82	33,508.00
CD24	CD24 molecule	4,273.60	6,136.86	5,046.04
ncRNA	Non-coding RNA	1,176.23	5,467.23	16,500.49
ncRNA	Non-coding RNA	685.90	5,428.94	16,401.81
LTF	Lactotransferrin	8,142.20	4,414.17	3,811.85
FTL	Ferritin, light polypeptide	2,715.15	4,262.47	2,882.36
B2M	Beta-2-Microglobulin	2,963.40	3,644.58	1,486.42
ncRNA	Non-coding RNA	3,098.27	3,392.18	1,018.07
ncRNA	Non-coding RNA	582.53	3,137.07	14,389.45
GLYCAM1	Glycosylation dependent cell adhesion molecule 1	2,187.31	2,878.29	4,108.55
SPP1	Secreted phosphoprotein 1	1,816.97	2,764.59	1,780.25
ncRNA	Non-coding RNA	459.06	2,539.38	7,970.79
FTH1	Ferritin, heavy polypeptide	1,774.86	2,505.45	3,398.50
TPT1	Tumor protein, translationally-controlled 1	2,736.16	2,347.90	2,982.91

The most abundant transcripts (Tables 1, 2 and 3) were similarly expressed among the three sample types, with a few notable exceptions. The milk fat fraction contained an abundance of mRNA for perilipin 2, a protein that coats lipid droplets [49] and is involved in milk fat globule formation [50-54]. Surprisingly, non-coding RNAs were also among these top transcripts and appeared to be enriched in milk fat relative to other sample types. Further inspection of these non-coding RNAs by BLAST against the NCBI database revealed homology to human ribosomal RNAs.

### Correlation of the milk and mammary transcriptomes

**Figure 7:**
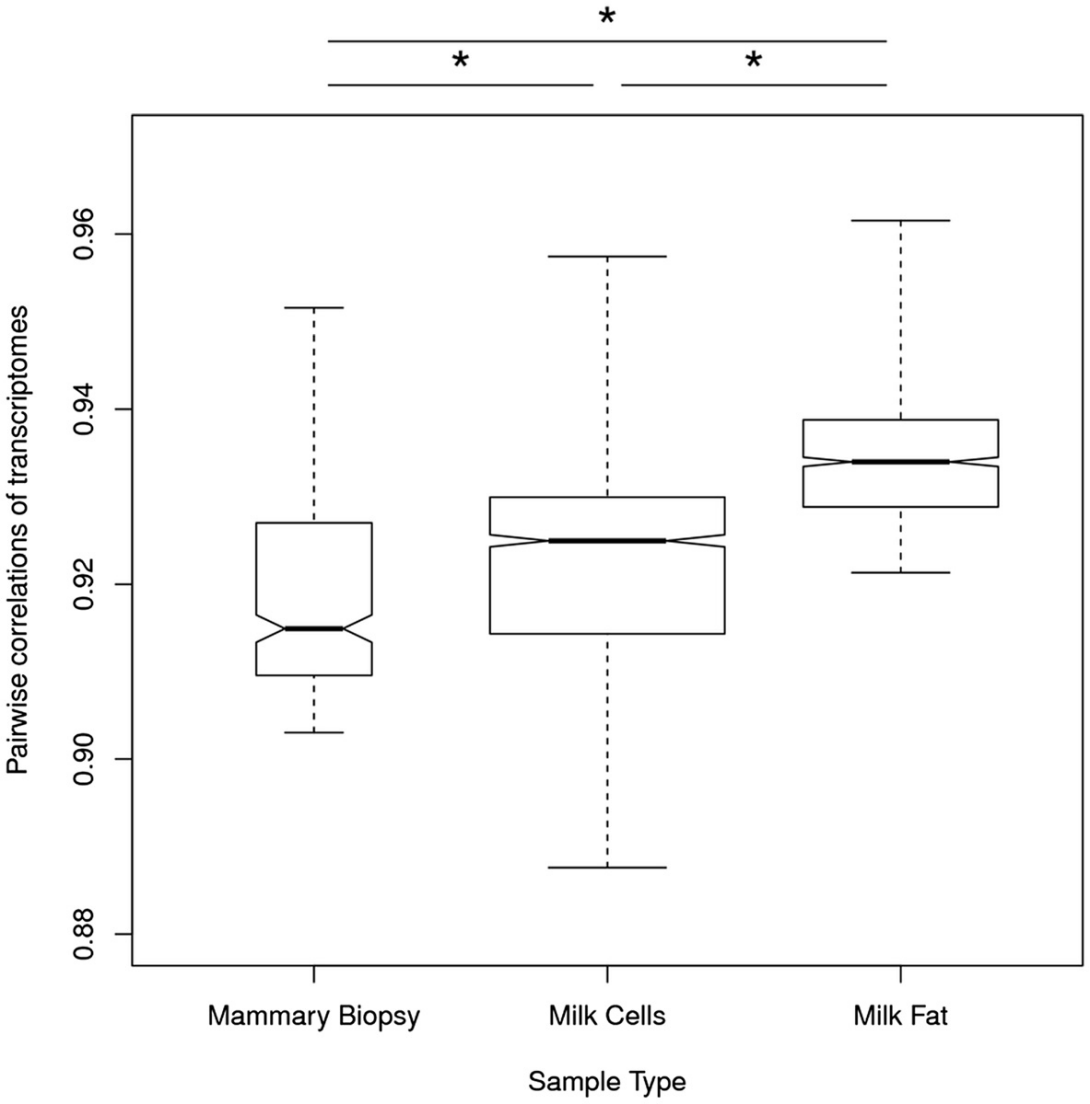
**Transcriptome homogeneity within sample types.** Transcriptomes derived from samples of the same type (mammary biopsy, milk cells, or milk fat) were compared with other samples of the same type using a Spearman’s correlation. The boxplot summarizes the findings by sample type. The upper and lower bounds of each box denote the first and third quartiles of the data, the dark horizontal line within each box denotes the median value, and the whiskers extend to the minimum and maximum values. The width of each box is proportional to the square root of the samples size. Asterisks denote statistical significance (*p* < = 0.05).

We hypothesized that milk fat samples, if dominated by exosomal RNA arising from milk-producing cells, would yield transcriptomes with greater similarity to each other compared with other sample types with more heterogeneous sources of RNA. To test this hypothesis, a Spearman’s correlation for all gene expression data was computed between every possible pair of macaque samples. Samples from the same tissue type were highly correlated with each other (Figure 7). Based on *t*-tests, the transcriptomes derived from milk fat were more highly correlated with each other (mean, 0.934) than those derived from mammary biopsy (mean, 0.919; *p* = 2.39e-69) or milk cell samples (mean, 0.922, *p* = 1.22e-145). The slightly higher correlation of milk cell samples with each other, compared with mammary biopsy samples, was also significant (*p* = 1.47e-05). The non-overlapping notches of the boxplots also suggest that the medians were significantly different [55]. In all cases, the transcriptome derived from each sample was highly similar to samples of the same type with the highest consistency occurring among milk fat samples.

**Figure 8:**
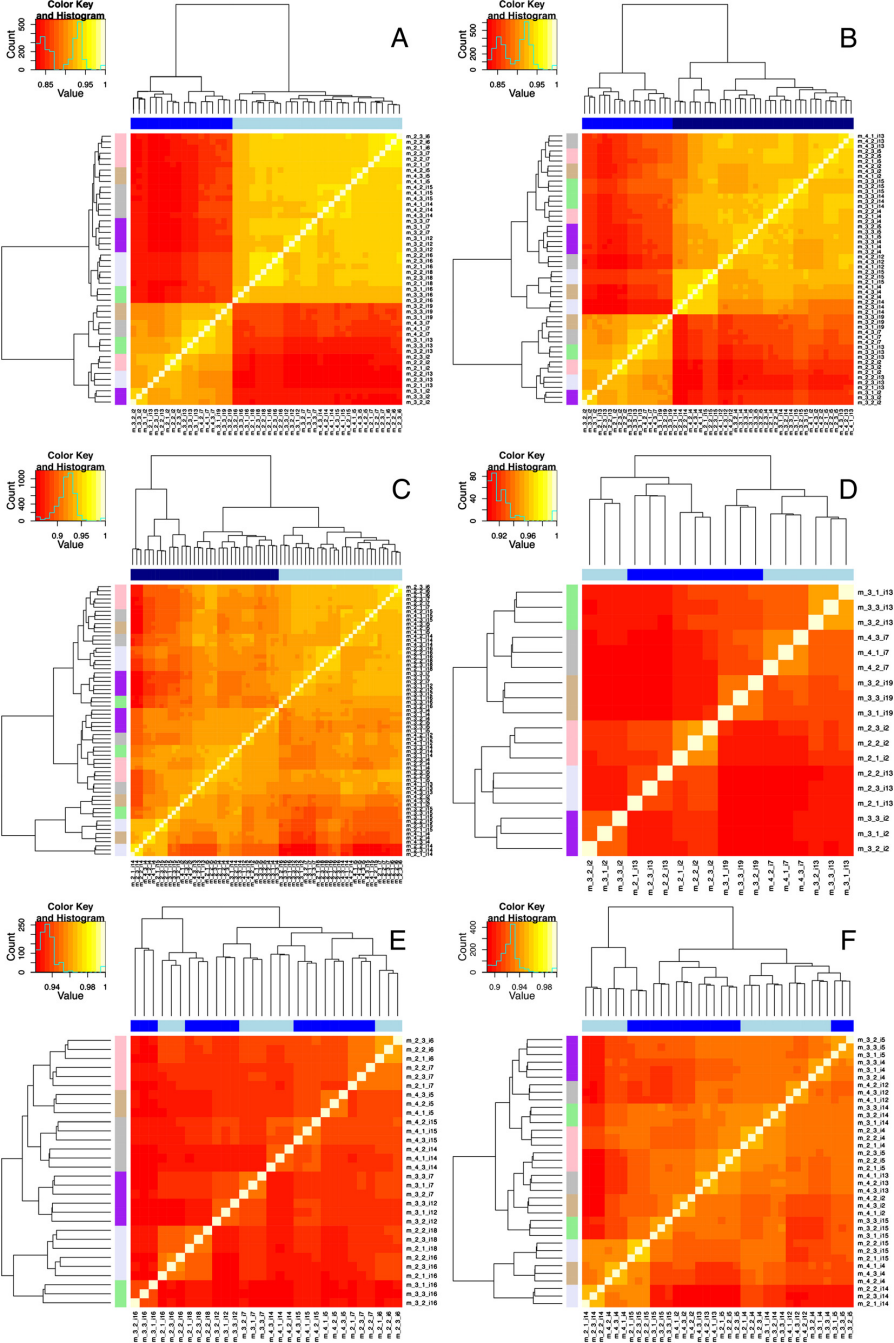
**Correlation of milk and mammary transcriptomes.** Each square in the heatmap represents the pairwise correlation of the sample listed in the row compared with the sample listed in the column. The shades of blue across the top of each heatmap correspond to sample type. The colors on the left side of each heat map correspond to each individual monkey. Both biological and technical replicates are shown. All technical replicates appear as clustered triplets in the dendograms. **(A)** Milk fat vs mammary biopsy samples; dark blue denotes biopsy, light blue denotes milk fat. **(B)** Milk cells vs mammary biopsy samples, dark blue denotes milk cell, light blue denotes milk fat. **(C)** Milk cells vs milk fat samples, dark blue denotes milk cell, blue denotes biopsy. **(D)** Mammary biopsy samples at day 30 and day 90. **(E)** Milk fat samples at day 30 and day 90. **(F)** Milk cells samples at day 30 and day 90. **(D–F)** Dark and light blue distinguish day 30 and 90 samples.

We next investigated the similarity of whole transcriptomes derived from the same animal, but different sample types (milk fat, milk cell, or mammary biopsy). To compare whole transcriptomes among sample types in the same animal, Spearman’s correlations were again computed and used to cluster the samples by similarity (Figure 8). Biopsy and milk samples clustered distinctly from each other. However, some milk cell samples were more similar to milk fat samples than they were to other milk cell samples. Although all milk fat samples from the same macaque clustered together, this was not true for all milk cell samples. Specifically, some milk cell samples taken from the same macaque, but at different time points, clustered more with samples from a different macaque. Whole transcriptomes derived from different time points of the same sample type did not cluster together (Figure 8). Other factors such as inter-individual or sampling differences created greater transcriptome variation than day of lactation. Among milk fat samples, but not milk cell samples, transcriptomes from the same macaque clustered together regardless of time point. Based on *t*-tests, the transcriptomes derived from milk fat samples from the same animal were more highly correlated with each other (mean, 0.935) than those derived from milk cell samples from the same animal (mean, 0.922, *p* = 5.625e-05). In summary, these correlation analyses suggest a slightly greater heterogeneity among samples of milk cells compared with milk fat.

### Differentially expressed genes in the milk and mammary transcriptomes

RNA-Seq data is inherently heteroskedastic in that abundantly expressed genes have greater variance in expression than low abundance genes. Therefore, we applied a variance stabilizing transformation using the DESeq package in R before testing for differential expression (see Methods). Differentially regulated genes (*p* < 0.05 and > 3-fold change) were identified among macaque milk fat, macaque milk cell, and macaque mammary biopsies. There were 2681 genes that were differentially regulated in mammary tissue and milk cells, where 1744 genes were expressed more in mammary tissue and 937 were higher in milk cells. These gene lists were tested for enrichment of clusters for biological process GO terms.

Transcripts upregulated in mammary tissue, relative to milk cells, included those associated with vasculature development, cell morphogenesis, regulation of cell mobility, cytoskeleton organization, wound healing, response to hormone stimulus, and regulation of cell adhesion. A complete list is provided in Additional file 4. These GO terms are consistent with those expected of the previously described heterogeneous cell types in mammary tissue functioning during lactation.

Transcripts upregulated in milk cells, relative to mammary tissue, were associated with chemotaxis, defense response, phosphorylation, calcium homeostasis, membrane organization and vesicle-mediated transport, apoptosis, regulation of interleukin-12 production, regulation of GTPase activity, regulation of biosynthetic processes, and regulation of cytokine production. Enrichment for these GO terms in milk cells highlighted that these cells are a heterogeneous population of immune and epithelial cells, as is widely accepted [56]. The “apoptosis” signature further suggested that some of these cells were dying, consistent with the appearance of dead cells in milk [57].

A comparison of mammary tissue and milk fat revealed 3174 genes that were differentially regulated. Of these, 2505 were higher in mammary tissue, and 669 were higher in milk fat. Upregulated genes in mammary tissue were associated with the same functions as described above. However, additional functions that were enriched in mammary tissue relative to milk fat included immune-specific functions, such as thymic T-cell selection, regulation of immune system processes, and regulation of acute inflammatory responses (Additional file 4). These findings confirmed that milk fat, relative to mammary tissue, was depleted of immune cells.

For the remaining 669 genes that were upregulated in milk fat relative to mammary tissue, there were no significant biological process GO terms that were enriched. After expanding the enrichment analysis to include all annotation types, three annotation clusters were significant—mitochondrion, oxidative phosphorylation (*p* = 1.1E-8), methyltransferase (*p* = 0.003), and intracellular membrane-enclosed lumen (*p* = 0.004). The intracellular membrane-enclosed lumen refers to an “enclosed volume within a sealed membrane or between two sealed membranes.” The RNA extracted from milk fat is theoretically derived from crescents of cytoplasm trapped between the two lipid bilayers of a double membrane surrounding the fat globule. These results confirmed that the milk fat was enriched with RNA derived from crescents of cytoplasm.

On comparing milk cells with milk fat, there were 1106 genes that were differentially regulated. Of these, 1050 genes were more abundant in milk cells, and 56 were higher in milk fat. Genes that were upregulated in milk cells, relative to milk fat, were involved in defense response, chemotaxis, regulation of the immune system, regulation of cytokine production, activation of leukocytes and lymphocytes, and other immune-related functions. “Apoptosis” was similarly enriched among milk cell transcripts (Benjamini *p* = 0.0004), suggesting that the milk cell pellet contained some dying cells. There were no significantly enriched GO terms associated with transcripts that were more abundant in milk fat.

Transcriptomes were also compared by lactation stage for each sample type. In mammary tissue, milk fat, and milk cell samples, there were 81, 48, and 247 genes, respectively, that were differentially regulated between day 30 and day 90. GO analysis was not fruitful due to the low number of genes in these lists. Consistent with the correlation analyses, stage of lactation appeared to have a larger impact on the milk cell transcriptome compared with other sample types.

Overall, the GO analyses supported that mammary tissue was a heterogeneous mixture of epithelial and stromal cells, milk cell fractions were a heterogeneous mixture of immune cells and epithelial cells, and that milk fat fractions were enriched with RNA of mammary epithelial cell origin.

## Discussion

Even with the guidance of lactation consultants, some mothers are unable to produce enough milk for their infants. This problem is pervasive in the U.S., with nearly 90% of mothers failing to meet the recommended duration of exclusive breastfeeding. While a variety of cultural, social, and economic factors influence breastfeeding duration, at least some of the inability to produce enough milk is expected to have a biological basis. In 2011, the U.S. Surgeon General issued a Call to Action to understand the causes of insufficient milk supply [58]. A non-invasive assay is much needed to identify and develop treatments for biological and physiological sources of insufficient milk production in lactating mothers. In the current study, we used a non-human primate model to validate whether a non-invasive milk-based assay could be used to track gene expression in milk-producing cells. When milk fat is secreted, cytoplasmic crescents containing RNA from the milk-secreting cell are carried into the milk. We found that rhesus macaques, like humans, released cytoplasmic crescents of RNA into their milk and that the RNA therein was representative of RNA from milk-producing mammary epithelial cells.

When the milk is separated into fractions, the fat layer is expected to contain the RNA from cytoplasmic crescents that were secreted by milk-producing mammary epithelial cells. Evidence for the use of this exosomal RNA extracted from milk fat as a replacement for mammary biopsies includes microscopic study and transcriptomic analysis. Patton and Huston [22] provided visual evidence of cytoplasmic crescents using fluorescence microscopy and AO. We extended their work using advanced imaging methods, whole slide scanning, and a quantification algorithm to determine the exosomal and cellular sources of RNA in milk. Our transcriptome results also confirmed the presence of cytoplasmic crescents in macaque milk, where the transcriptome from the milk fat fraction was enriched in mammary epithelial-specific transcripts and depleted of stromal and immune cell-specific transcripts. Given that macaque milk has a higher percentage of cellular RNA and lower percentage of exosomal RNA in whole milk, it can be surmised that human milk would be even more amenable to isolation of cytoplasmic crescent RNA from milk fat. Fortunately, our study confirmed the validity of prior work in which RNA was extracted from human milk fat to assess the transcriptome of milk production [19-21,26].

A limitation of our study is that all sample types—mammary tissue, milk fat, and milk cells—were confounded by the presence of multiple cell types (Figure 5). In particular, it is nearly impossible to discern whether the transcriptomic signatures of epithelial origin in milk fat arise from cytoplasmic crescents secreted by milk-producing cells *in vivo* or from exfoliated epithelial cells. However, the greater abundance of KRT8, and not KRT5, suggests that the milk fat fraction uniquely contained RNA arising from milk-producing cells, rather than other epithelial cell types. Also, the presence of apoptotic signatures in the milk cell fraction, but not the milk fat fraction, further suggests that the epithelial signatures in the milk fat fraction derived from cells *in vivo*, rather than dying exfoliated cells.

Each sample type—mammary tissue, milk fat, and milk cells—has its own place in research settings. The advantages and disadvantages of each sample type as an RNA source are summarized in Table 4. Mammary tissue will be required when the nuclei of mammary epithelial cells is desired, in which case such cells can be isolated through enzymatic dissociation or laser capture micro-dissection. Milk fat samples provide RNA from the cytoplasm of mammary epithelial cells without the invasiveness, cost, or inconvenience of a mammary biopsy. In macaques and humans [21], the collection of RNA from the milk fat layer has several advantages, including abundant mammary epithelial RNA, minimal stromal or immune cell RNA, and high reproducibility when samples are taken from the same subject. The primary disadvantage of using RNA from milk fat samples appears to be its vulnerability to degradation, although in our study, this was only observed in human samples.

**Table 4:** Advantages and disadvantages of sample type as RNA source

**RNA source**	**Advantages**	**Disadvantages**
Mammary tissue	Can isolate specific cells	Invasive
Milk cells	Non-invasive	Heterogeneous cell population
	Epithelial cell signature	Some dying cells
	Relatively stable RNA	Sensitive to infection, inflammation
Milk fat	Non-invasive	RNA vulnerable to degradation
	Epithelial cell signature	
	No/low stromal or immune cell signature	

Degradation of RNA due to processing time was not significantly different among macaque samples, despite processing delays similar to those for the human collections. The different effect of processing time between human and macaque samples may reflect differences in sample handling due to the involvement of human participants versus the controlled conditions of the primate center. Intriguingly, there was no difference in RNA degradation in macaque milk collected after 10 min or 4 hr of accumulation, suggesting that degradation of milk fat RNA may not occur within the mammary gland during accumulation periods as long as 4 hr. This is unexpected because the mRNA degrades quickly post-collection; in our study, differences in transport and processing times as small as 30 to 60 min negatively affected RNA quality.

As could be expected, cells in macaque milk appeared to be a heterogeneous population of both immune and epithelial cells. The enrichment of the “apoptosis” GO term among transcripts suggested that at least some of these cells were dying, as was observed in other studies of exfoliated mammary epithelial cells [59]. There was also a question of reproducibility given the lower correlations of milk cell transcriptomes in samples from the same subject. RNA derived from milk cells was also slightly more sensitive to lactation stage. However, RNA from cells in milk was more resistant to degradation. Thus, in field conditions in which fast collection and processing of a milk sample to preserve RNA is not possible, it would be preferable to use RNA from cells in whole milk instead of milk fat. However, attention must be given to conditions such as infection, non-infectious inflammation, and stage of lactation that can perturb populations of cells in milk.

The presence of multiple epithelial cell types in milk cell fractions opens the door to potential breast cancer diagnostics in lactating women. This is particularly important because breast cancers diagnosed within five years of pregnancy have a poorer prognosis [60] with a peak mortality rate associated with cancers diagnosed at 4 to 7 months postpartum [61-63], precisely when women may still be breastfeeding. Our analysis of cell marker-associated transcripts from cells in milk (Figure 5) suggests that populations of epithelial cells in milk are heterogeneous and are not just milk-secreting epithelial cells. For the purposes of diagnostics, access to additional cell types in the pelleted milk cells is advantageous because breast cancers can arise in several different cell types. Along these lines, Brown et al. [27] described DNA methylation patterns in epithelial cells extracted from milk cell pellets as potential biomarkers of breast cancer. Transcriptional profiles of pelleted milk cells from lactating women with breast cancer offer another avenue for biomarker discovery.

Our findings for humans and macaques may not be readily extendible to other species such as the dairy cow given that the incidence of cytoplasmic crescent formation is species-specific. Compared with humans and macaques, cows secrete fewer cytoplasmic crescents of RNA into their milk [22,23]. Thus, one would expect a lower percentage of mammary epithelial cell-derived RNA in the fat fractions of milk from cows compared with primate milks. Additional studies involving bovine tissues and milk samples will be needed to determine the validity of this assay for dairy science.

Our observation that humans produced cytoplasmic crescents at a slightly higher rate than rhesus macaques begs questions as to its functional significance. Three proteins are believed to coordinate the formation of milk fat globules—xanthine oxidoreductase, butyrophilin, and adipophilin [51]. A phylogenetic analysis of these genes and their regulatory regions together with quantitation of cytoplasmic crescent formation may yield the genetic basis for variation in cytoplasmic crescent incidence. Given that human milk-producing cells lose such a large amount of cytosol during milk secretion, one might speculate that this serves an important biological function. One possible function would be the transfer of microRNA from mother to offspring [64,65]. Arguments against important functionality of cytoplasmic crescents include the large variation among individuals (Figure 2A) and the high incidence of variation in certain non-human species such as goats [24].

This study presents evidence for the use of lactating rhesus macaques as a model for human lactation. We confirmed that cytoplasmic crescents are secreted in macaque milk, can be isolated in the milk fat fraction, and that ample sequence-quality RNA can be obtained from macaque milk fractions. Both the milk and mammary-derived transcriptomes suggested that the process of milk production in macaques matches expectations of biological function. Developmental and hormonal responses of mammary glands in macaques show a high degree of similarity to those of the human breast [66]. We confirmed that histology of the mammary glands in rhesus macaques resembles normal human mammary tissue during lactation. Recent studies also validated the infant macaque as a model for human infant metabolism [67,68]. Thus, macaque mother-infant dyads provide a unique model to study milk production and consumption, and the consequences of early life experiences.

## Conclusions

Milk sampled from humans and macaques provided an accurate and convenient means to assess the transcriptome of milk-producing mammary epithelial cells. Total RNA extracted from macaque milk provided an even better representation of RNA from mammary epithelial cells than did RNA from whole mammary tissue. Mammary epithelial-specific transcripts were more abundant in milk fat samples, whereas non-epithelial transcripts associated with stromal cells were more abundant in biopsied mammary tissue. For the study of the transcriptome of milk-producing cells in primates, we suggest that milk trumps mammary tissue because RNA from milk fractions, especially milk fat, is highly enriched with transcripts specifically arising from milk-producing cells. The findings from rhesus macaques should extend to humans, who secrete cytoplasmic crescents into their milk at slightly higher rates and with fewer somatic cells. In summary, we validated the use of RNA extracted from human and macaque milk and provided evidence to support the use of lactating macaques as a model for human lactation.

## Availability of supporting data

The raw sequencing data and processed data have been deposited in NCBI’s GEO database, accession GSE49765. Detailed protocols, algorithms, and processed data are provided as Additional file 1, Additional file 2, Additional file 3 and Additional file 4.

## Competing interests

The authors declare that they have no competing interests.

## Authors’ contributions

DGL designed research; DGL, RCH, SRH, KH, JTS, FV, KAS, JWSL, AI-T, and PIG conducted research; all authors analyzed data; DGL and RCH wrote the paper. All authors read and approved the final manuscript.

## Supplementary Material

Additional file 1 Milk processing protocols for human and macaque milk. Click here for file

Additional file 2 An explanation of the algorithm used to quantify globules and crescents by the Globulator software. Click here for file

Additional file 3 FPKM levels for each macaque transcript and each condition. Click here for file

Additional file 4 Complete gene ontology results. Click here for file
