# Supplementary material for: Sequencing the transcriptome of milk production: milk trumps mammary tissue
Source: BMC Genomics. 2013 Dec 12;14:872. doi: 10.1186/1471-2164-14-872 (PMC3871720; doi:10.1186/1471-2164-14-872)
Supplement: Additional file 1 — Milk processing protocols for human and macaque milk. [file 1471-2164-14-872-S1.docx]

Human and Macaque Milk Processing Protocols

The following detailed protocols describe how to visualize and stabilize RNA from human and macaque milks. Cytoplasmic crescent visualization was adapted from Patton and Huston^1^. Milk fat isolation was adapted from the Haymond Lab at Baylor College of Medicine^2^. Milk cell isolation adapted from the Medrano Lab at UC Davis^3^.

Protocol: MFG Crescent Visualization

For immediate viewing:

1. Add 1 mL of 0.1 M phosphate buffer pH 6.8 to 1 mg acridine orange to make a 0.1% acridine orange solution.
2. For human milk,

Aliquot 1 mL of whole milk to a new tube

Add 100 uL of 0.1 % acridine orange solution

1. For macaque milk,

Aliquot 100 uL of WHOLE milk to a new tube

Add 10 uL of 0.1 % acridine orange solution

1. Incubate (wait) 5 minutes
2. Use a disposable pipette to put one drop on a slide. Seal coverslip with nail polish. Label slide.
3. View with fluorescent microscope. When acridine orange associates with RNA (cytoplasmic crescent), the excitation maximum is 460 nm (blue) and the emission maximum is 650 nm (red). When acridine orange associates with DNA (nucleated cell), the excitation maximum is 502 nm and the emission maximum is 525 nm (green).

Protocol: RNA Isolation from Whole Macaque Milk

Keep all samples on ice. Use Biosafety Level 2 procedures.

1. Turn on the centrifuge and set to 4 C, ~2311g. Pre-weigh 2mL “milk fat” tubes.
2. WEIGH all whole milk samples and RECORD.
3. Gently swirl/rock to get a good mixture, then use pipet-aid with serological tips to determine VOLUME and RECORD.
4. If whole milk is 10mL or less, transfer all to centrifuge tube. If more than 10mL, divide among centrifuge tubes.
5. Centrifuge the whole milk at 2311g for 10 min at 4 C.
6. When milk is done spinning, collect fat layer using a sterile spatula and transfer into 2mL “milk fat” tubes.
7. WEIGH and RECORD the 2mL tubes with fat.
8. Add 1.5mL Trizol to each 2mL tube.
9. From centrifuge tubes, remove skim fraction using a pipette.
10. Add 2-3 mL cold PBS to each centrifuge tube and gently pipette up and down until pellet is dislodged and pool all samples **of the same animal**.
11. Centrifuge cell suspension 2311g for 10min at 4 C.
12. Decant supernatant of the centrifuge tube.
13. Add 1mL TRIzol to pellet, pipet up and down to lyse cells, transfer.
14. Move all samples from ice to -80 C until further processing to extract and purify RNA.
15. Sterilize spatulas for future RNA work.

Protocol: RNA Isolation from Whole Human Milk

Keep all samples on ice.

1. Turn on the centrifuge and set to 4 C, ~2311g.
2. Remove foil from autoclaved glass beaker, weigh and record.
3. Remove foil from autoclaved glass cylinder. GENTLY pour all milk samples into the cylinder and record the volume.
4. GENTLY pour all milk sample into the beaker. Weigh and record.
5. GENTLY swirl the whole milk in the beaker to get a good mixture, then aliquot from the beaker using a pi-pump or pipet-aid with 10mL glass serological tips: aliquot 10 mL volumes into up to four 15 mL centrifuge tubes and keep on ice.
6. Centrifuge the four tubes at 2311g for 10 min at 4 C.
7. Weigh and record the empty 2mL tubes that are labeled for milk fat.
8. When milk is done spinning, transfer supernatant fat layer from each tube using sterile spatula to 2mL tubes labeled for milk fat.
9. Weigh and record the 2mL tubes with fat.
10. IN THE HOOD, add 1mL Trizol to each 2mL tube, homogenize by shaking/vortexing/pipetting, incubate for 5 min, then store at -80 C.
11. Return to the centrifuge tubes, carefully decant the liquid of each
12. Add 2-3 mL PBS to each of the four centrifuge tubes and gently pipette up and down until pellet is dislodged and pool all four samples into one tube.
13. Add water to a second centrifuge tube and adjust volume using the balance.
14. Centrifuge the two tubes at 2311g for 10min at 4 C.
15. Decant supernatant of the centrifuge tube. Wipe residual fat off of tube sides with a tissue. (We used a kimwipe on the end of a sterile spatula)
16. IN THE HOOD, add 1mL TRIzol to the pellet and pipet up and down to lyse cells. Transfer to 2mL tube labeled for milk cell fraction.
17. Move all samples from ice to -80 C until further processing to extract and purify RNA.
18. Sterilize glassware and spatulas for future RNA work.

1. Patton S, Huston GE. Incidence and characteristics of cell pieces on human milk fat globules. Biochim Biophys Acta. 1988 May 12;965(2-3):146-53. PubMed PMID: 3365450.

2. Maningat PD, Sen P, Sunehag AL, Hadsell DL, Haymond MW. Regulation of gene expression in human mammary epithelium: effect of breast pumping. J Endocrinol. 2007 Dec;195(3):503-11. PubMed PMID: 18000312.

3. Wickramasinghe S, Rincon G, Islas-Trejo A, Medrano JF. Transcriptional profiling of bovine milk using RNA sequencing. BMC Genomics. 2012 Jan 25;13:45. doi: 10.1186/1471-2164-13-45. PubMed PMID: 22276848; PubMed Central PMCID: PMC3285075.
