# Supplementary material for: Sequencing the transcriptome of milk production: milk trumps mammary tissue
Source: BMC Genomics. 2013 Dec 12;14:872. doi: 10.1186/1471-2164-14-872 (PMC3871720; doi:10.1186/1471-2164-14-872)
Supplement: Additional file 2 — An explanation of the algorithm used to quantify globules and crescents by the Globulator software. [file 1471-2164-14-872-S2.docx]

**Globulator Algorithm**

**
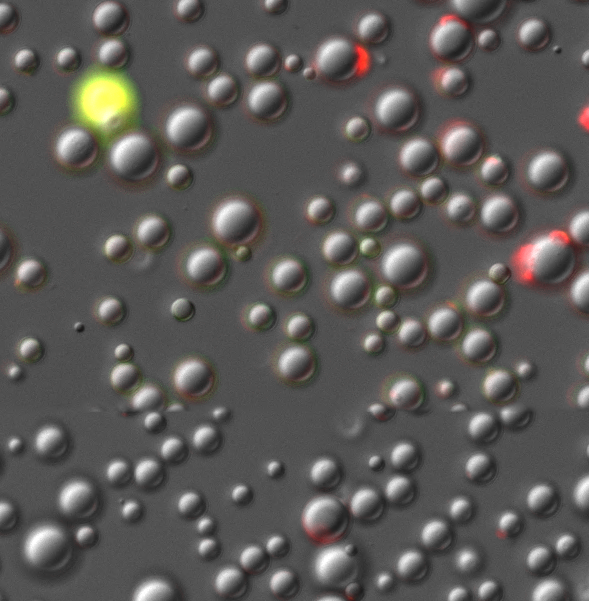

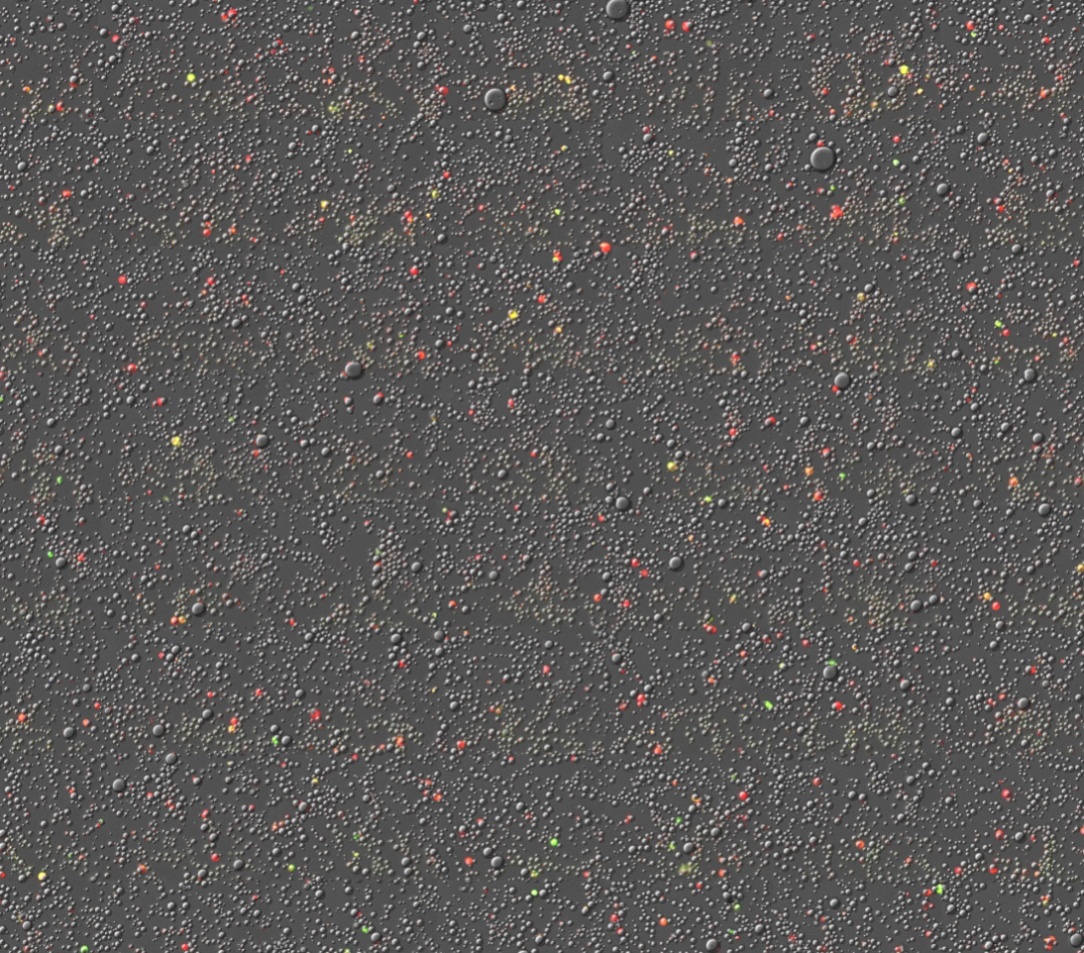
**

**B**

**A**

Globule with Crescent

Crescent

Globule without Crescent

Nucleated cell

**C**

**D**

**
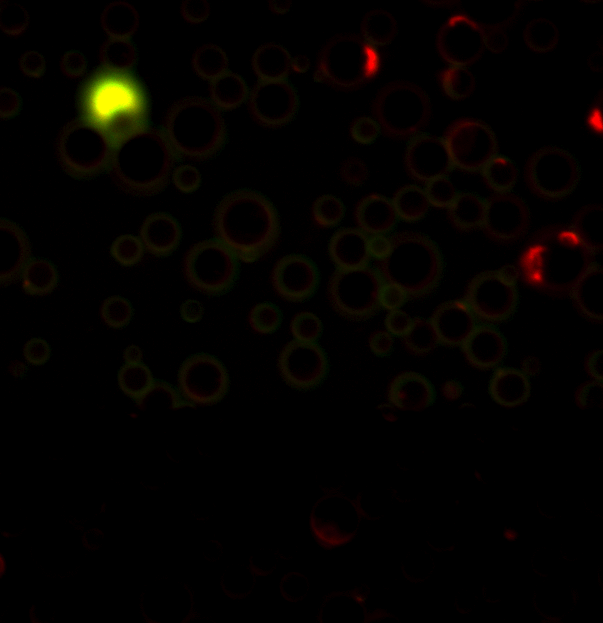

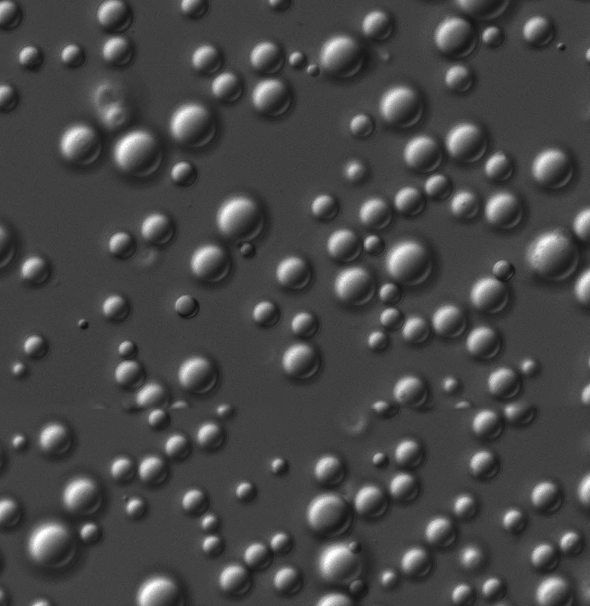
**

**1: Input slides of whole milk stained with Acridine Orange.**

Each whole milk slide contains three channels: 1) a differential interference contrast (DIC) image to view the fat globules, which look like gray bubbles, 2) a fluorescence channel for AO-RNA, and 3) a second fluorescence channel for AO-DNA. When acridine orange associates with RNA, the emission maximum is 650 nm (red). When AO associates with DNA, the emission maximum is 525 nm (green). Therefore, areas containing only RNA (e.g. cytoplasmic crescents) look red, only DNA look green, and both RNA and DNA (e.g. nucleated cells) look yellow. **(A)** An example composite whole milk slide which contains >20000 globules, crescents, and nucleated cells. **(B)** Close-up of sample input slide, showing crescents, globules with and without crescents, and nucleated cells. **(C)** DIC channel showing fat globules. **(D)** Fluorescence channel containing AO-RNA and AO-DNA showing crescents (red) and a nucleated cell (yellow-green).

**2: Capturing globule/crescent locations**

ImageJ can be set to automatically measure the (X,Y) coordinates and area of a region containing a desired color range. **(A)** DIC channel containing milk fat globules. Globules appear as white-grey bubbles, which overlap with the grey background color, therefore ImageJ is set to only measure the white region of the globule. The measured white portion is marked with red color. **(B)** Black outlines show the measured regions. The resulting globule area is an incomplete circle, which is later corrected. **(C)** Fluorescent channel containing AO-RNA and AO-DNA. Crescents are red, therefore only regions with red color are measured. The measured crescent is marked here with white color. **(D)** Black outlines depict the locations of crescents. Nucleated cells are also measured by yellow-green color and calculated independently.

**A**

**C**


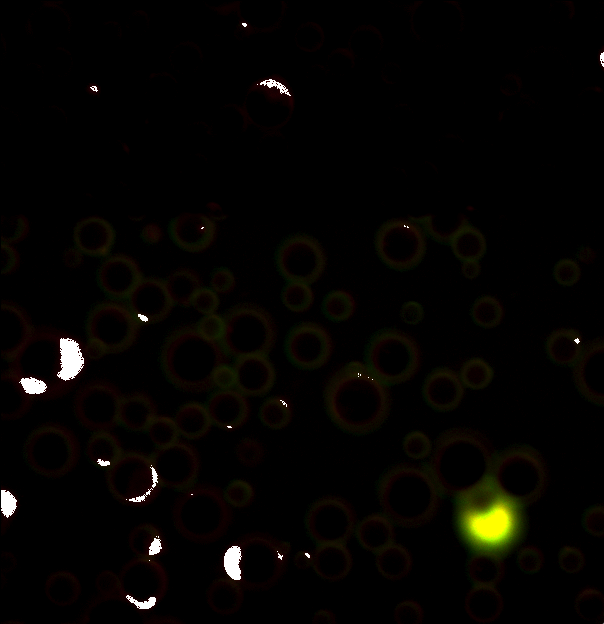

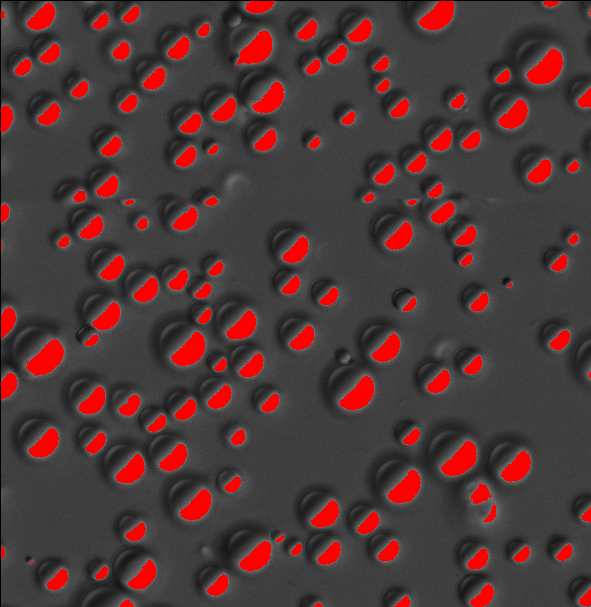


Measured Globule

**B**

**D**

Measured Crescent


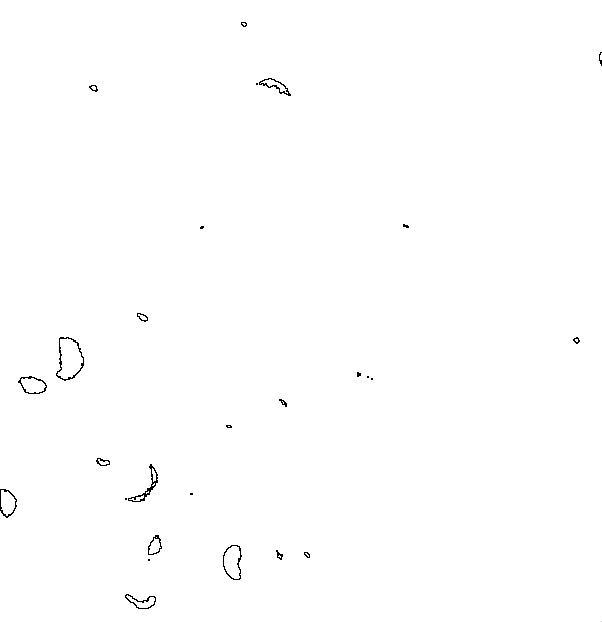

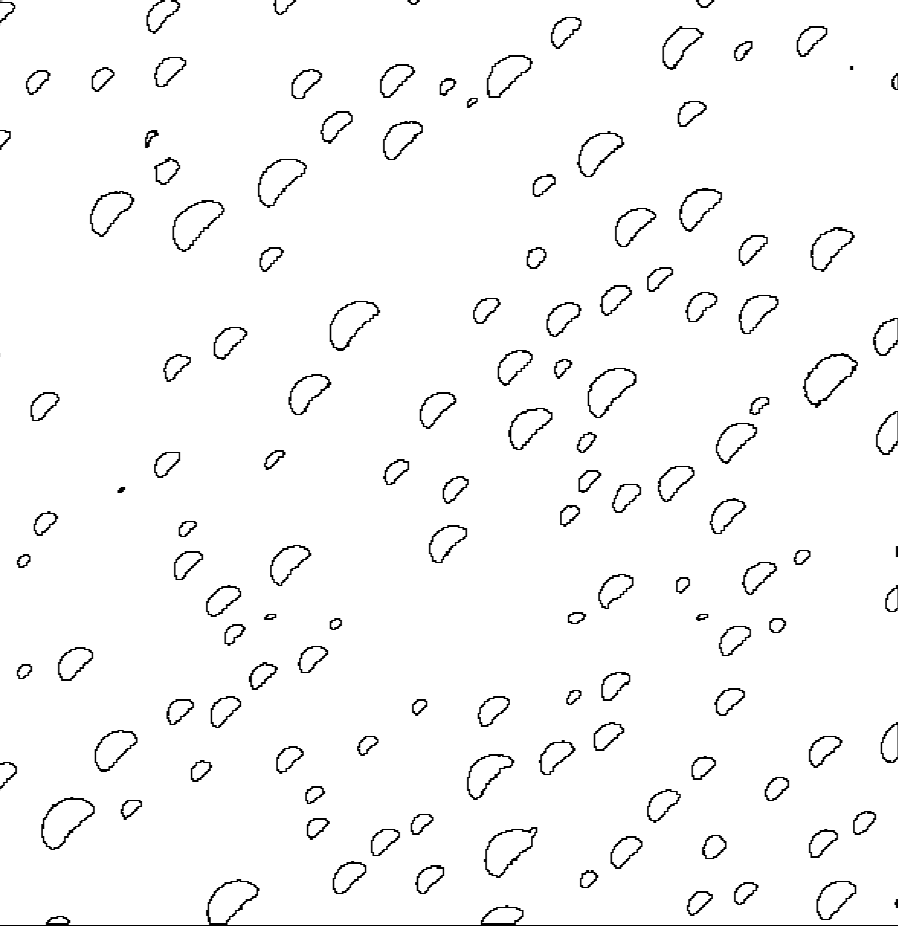


**C**

**B**

**A**


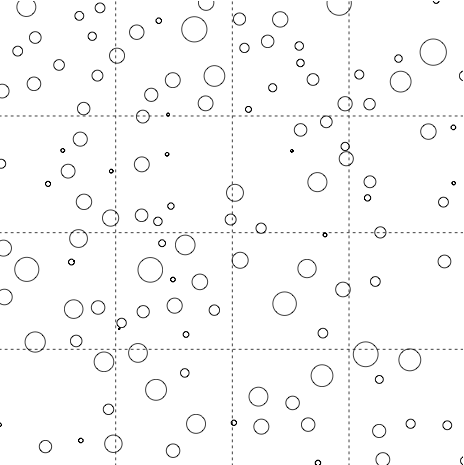

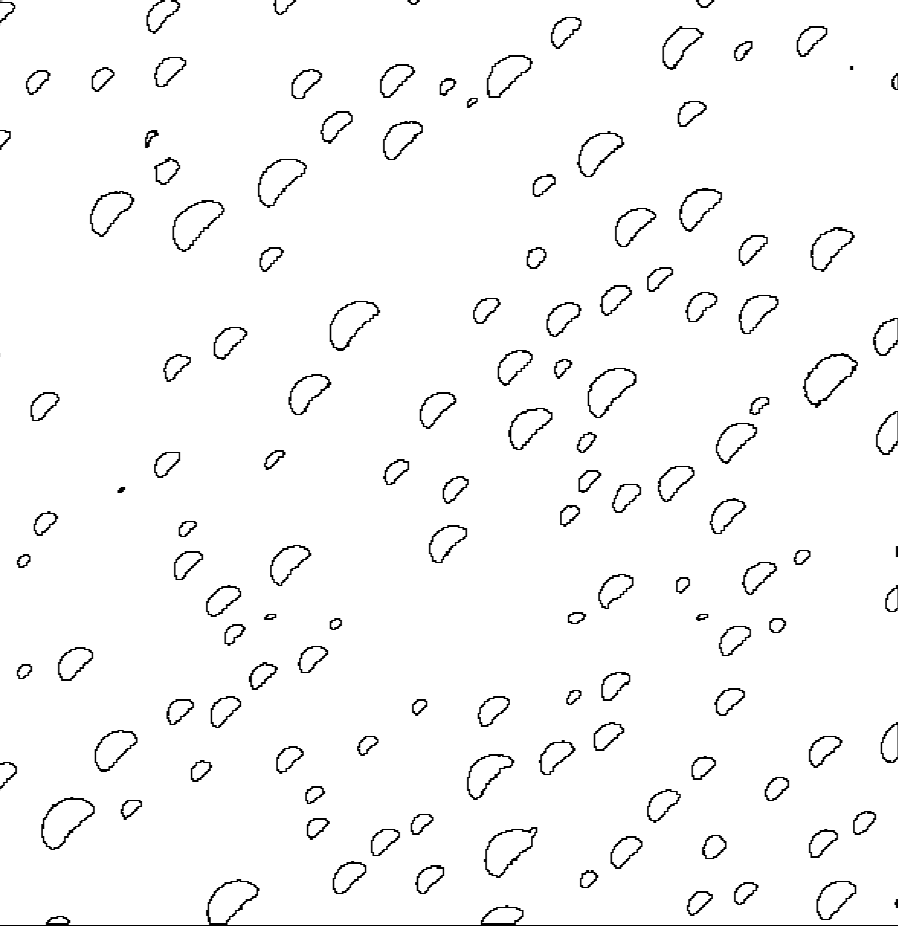

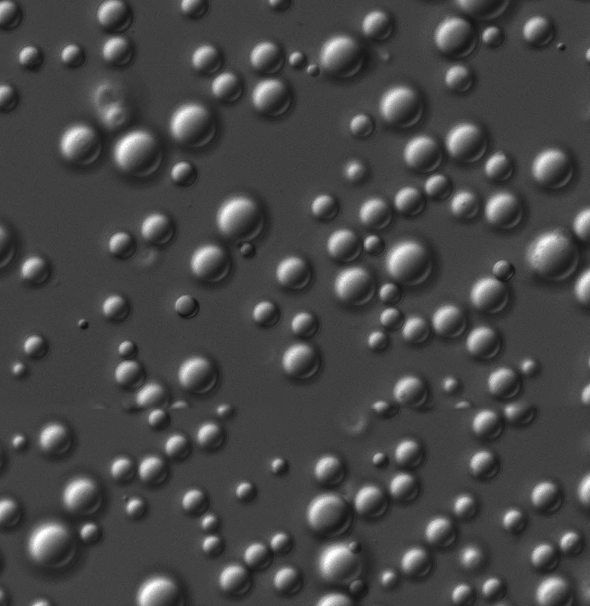


**3: (X,Y) coordinates and area adjustment of measured semi-circular globules.**  **(A)** Globule locations in the DIC image are **(B)** captured as semi-circles and then **(C)** corrected based on the assumption that the true globule shape is circular.

Ra

Rb

Rc

Dist AC

Dist BC

Blue solid line is

the real distance

**4: Algorithm to link each crescent with its globule.** Globules and crescents are independently measured, therefore each crescent needs to be linked with a globule. Crescents are assumed to be circular. Using a Pythagorean calculation, each crescent is linked with the closest globule. In this example, Crescent C is linked with Globule X.

**A**

**B**

**C**


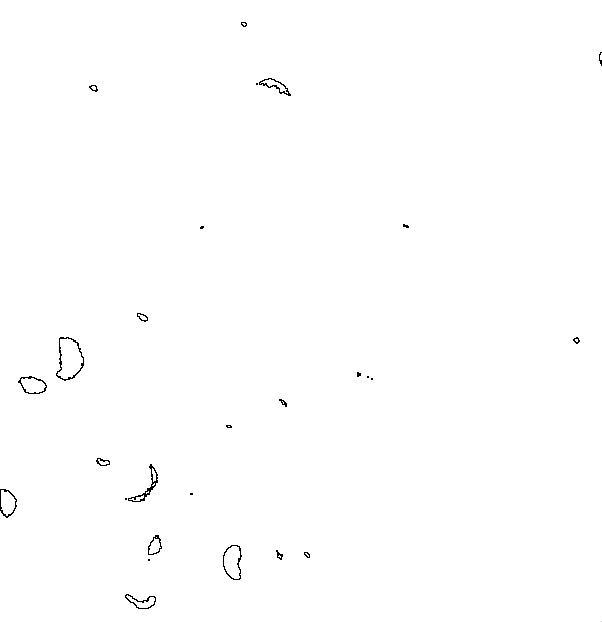

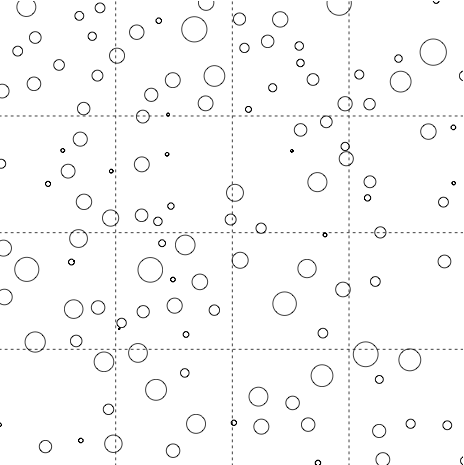

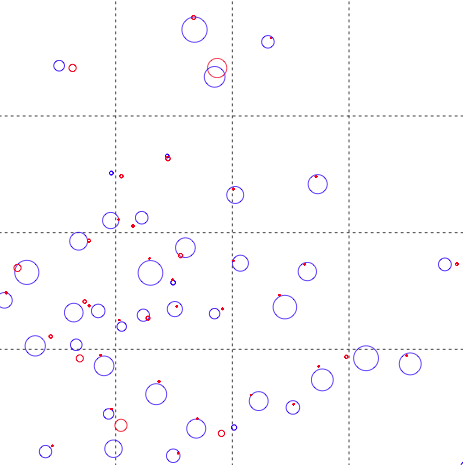


+

**5: Generation of images for manual validation.** A script was created to display globules and crescents identified by the Globulator software. This image was then manually compared to the original slide to assess accuracy. **(A)** Globule measurements. **(B)** Crescent measurements. **(C)** Crescents (red circles) linked with globules (blue circles).
