# Supplementary material for: Sequencing the transcriptome of milk production: milk trumps mammary tissue
Source: BMC Genomics. 2013 Dec 12;14:872. doi: 10.1186/1471-2164-14-872 (PMC3871720; doi:10.1186/1471-2164-14-872)
Supplement: Additional file 4 — Complete gene ontology results. [file 1471-2164-14-872-S4.docx]

**Summary of GO Analyses**

Separated by up/down change in expression, converted to human orthologs.

Ran DAVID functional clustering analysis with GO biological process ONLY.

Gene list: **highFC_up_Cells_vs_Bx (1370 DAVID IDs)**

**Upregulated in Bx relative to Cells*** Benjamini p-value

Vasculature development p = 1.7E-8

Skeletal system development p = 1.9E-10

Neuron differentiation / cell morphogenesis p = 1.4E-5

Cell motion, regulation of p = 9.2E-5

Cytoskeleton organization p = 5.4E-4

Tube development p = 2.9E-4

Wound healing p = 4.8E-6

Response to hormone stimulus p = 2.4E-2

Regulation of cell adhesion p = 4.2E-3

Response to vitamin p = 4.6E-3

Regulation of cell growth p = 1.2E-4

Regulation of cell morphogenesis p = 2.5E-3

Muscle organ development p = 5.9E-4

Pattern specification process** p = 8.5E-3

Negative regulation of biosynthetic process p = 3.3E=2

Female sex differentiation p = 4.3E-2

*Sorted by Enrichment Score (Highest first), only clusters with significant (Benj. p < 0.05) constituents listed

**Any developmental process that results in the creation of defined areas or spaces within an organism to which cells respond and eventually are instructed to differentiate.

Gene list:

| **highFC_up_Fat_vs_Bx** | **1965 DAVID IDs** |
| --- | --- |

**Upregulated in Bx relative to Fat*** Benjamini p-value

Cell adhesion, regulation of p = 4.4E-24

Cell motion, regulation of p = 4.1E-10

Vasculature development p = 1.2E-7

Response to wounding p = 6.5E-14

Chemotaxis p = 3.4E-6

Cytoskeleton organization p = 2.4E-4

Skeletal system development, morphogenesis p = 1.4E-7

Cell morphogenesis p = 1.0E-4

Tube development p = 1.8E-3

Response to vitamin p = 1.2E-4

Response to hormone stimulus p = 8.6E-3

Wound healing p = 7.9E-7

Pattern specification process p = 3.2E-3

Positive regulation of immune system process p = 1.0E-4

Regulation of cell growth p = 6.2E-3

Thymic T-cell selection p = 7.9E-3

Regeneration p = 2.1E-3

Muscle organ development p = 4.7E-3

Regulation of acute inflammatory response p = 8.1E-3

Positive regulation of cell communication p = 7.8E-3

Positive regulation of cell differentiation p = 5.6E-4

Tissue homeostasis p = 6.5E-2

Negative regulation of biosynthetic process p = 4.5E-2

Gene list:

| **highFC_up_Fat_vs_Cells** | **828 DAVID IDs** |
| --- | --- |

**Upregulated in Cells relative to Fat*** Benjamini p-value

Defense response p = 2.9E-35

Chemotaxis, cell motion p = 1.8E-20

Positive regulation of immune system p = 4.3E-22

Regulation of cytokine production p = 7.1E-13

Cell activation (leukocyte, lymphocyte) p = 5.0E-18

Regulation of response to external stimulus p = 4.3E-22

Cellular calcium ion homeostasis p = 1.4E-7

Response to LPS, bacterium p = 6.5E-6

Positive regulation of cell communication p = 8.9E-7

Positive regulation of cytokine biosynthetic process p = 1.4E-5

Lymphocyte differentiation p = 3.2E-7

Regulation of phosphorylation, de-phosphorylation p = 2.0E-7

Cell adhesion p = 4.6E-5

Apoptosis p = 4.1E-4

Regulation of adaptive immune response p = 1.0E-8

Response to glucocorticoid stimulus p = 3.6E-5

Regulation of hydrolase activity (lipase) p = 1.4E-3

Collagen catabolism p = 5.0E-3

Regulation of DNA binding p = 5.0E-3

Wound healing p = 1.5E-3

Positive regulation of transport, cytokine secretion p = 5.8E-5

Lymphocyte proliferation p = 4.7E-2

Phagocytosis p = 4.1E-4

Gene list:

| **highFC_down_Fat_vs_Cells** | **40 DAVID IDs** |
| --- | --- |

**Downregulated in Cells relative to Milk Fat*** Benjamini p-value

NONE SIGNIFICANT

Gene list:

| **highFC_down_Fat_vs_Bx** | **449 DAVID IDs** |
| --- | --- |

**Downregulated in Bx relative to Milk Fat*** Benjamini p-value

NONE SIGNIFICANT

Gene list:

| **highFC_down_Cells_vs_Bx** | **697 DAVID IDs** |
| --- | --- |

**Downregulated in Bx relative to Milk Cells*** Benjamini p-value

Chemotaxis, defense response p = 5.9E-6

Cell activation (leukocyte, lymphocyte) p = 1.4E-3

Phosphorylation p = 3.1E-2

Cellular cation homeostasis (calcium) p = 2.7E-3

Membrane organization, vesicle-mediated transport, endocytosis p = 2.5E-2

Apoptosis p = 4.2E-2

Regulation of interleukin-12 production p = 2.9E-2

Regulation of GTPase activity p = 1.0E-2

Positive regulation of biosynthetic process p = 1.6E-2

Positive regulation of cytokine production p = 9.6E-4
